# Caveolin-1α regulates primary cilium length by controlling RhoA GTPase activity

**DOI:** 10.1038/s41598-018-38020-5

**Published:** 2019-02-04

**Authors:** Laura Rangel, Miguel Bernabé-Rubio, Jaime Fernández-Barrera, Javier Casares-Arias, Jaime Millán, Miguel A. Alonso, Isabel Correas

**Affiliations:** 1 Department of Cell Biology and Immunology, Centro de Biología Molecular Severo Ochoa, Consejo Superior de Investigaciones Científicas and Universidad Autónoma de Madrid, Madrid, Spain; 2 Department of Molecular Biology, Universidad Autónoma de Madrid, Madrid, Spain

## Abstract

The primary cilium is a single non-motile protrusion of the plasma membrane of most types of mammalian cell. The structure, length and function of the primary cilium must be tightly controlled because their dysfunction is associated with disease. Caveolin 1 (Cav1), which is best known as a component of membrane invaginations called caveolae, is also present in non-caveolar membrane domains whose function is beginning to be understood. We show that silencing of α and β Cav1 isoforms in different cell lines increases ciliary length regardless of the route of primary ciliogenesis. The sole expression of Cav1α, which is distributed at the apical membrane, restores normal cilium size in Cav1 KO MDCK cells. Cells KO for only Cav1α, which also show long cilia, have a disrupted actin cytoskeleton and reduced RhoA GTPase activity at the apical membrane, and a greater accumulation of Rab11 vesicles at the centrosome. Subsequent experiments showed that DIA1 and ROCK help regulate ciliary length. Since MDCK cells lack apical caveolae, our results imply that non-caveolar apical Cav1α is an important regulator of ciliary length, exerting its effect via RhoA and its effectors, ROCK and DIA1.

## Introduction

The primary cilium is a non-motile organelle that protrudes from the cell surface of most mammalian cell types. The organelle derives from the basal body, which is the older of the two centrioles in the centrosome, and is made up of a nine-microtubule-doublet structure, called the axoneme, which is surrounded by a specialized ciliary membrane^1,2^. The primary cilium plays a crucial role as antennae for signal transduction in apparently disparate processes, such as photoreception and mechanosensation, and in a number of signaling pathways that are important for cell development, proliferation, differentiation and migration, such as those involving sonic hedgehog, Wingless/Int, and platelet-derived growth factor α^1,3–5^. Cilia dysfunction generates a broad spectrum of genetic disorders, collectively known as ciliopathies, that lead to cystic kidneys, retinal degeneration, obesity or mental retardation, among others^6–8^.

Given the importance of the primary cilium, its formation, length, structure and composition are tightly regulated. Primary cilia formation begins at cell cycle exit^9,10^. It has been proposed that primary ciliogenesis proceeds by two distinct pathways^11^. In cells of connective tissues, such as fibroblasts and chondrocytes, the process of primary cilium formation starts intracellularly with the docking of small cytoplasmic vesicles in the distal part of the mother centriole. These vesicles then fuse, generating a large ciliary vesicle that progressively expands, gradually becoming deformed by the elongation of a nascent axoneme. Finally, the ciliary vesicle is exocytosed and fuses with the plasma membrane, exposing the incipient cilium to the extracellular milieu in such a way that the membrane on the side of the vesicle facing the axoneme becomes the ciliary membrane. In contrast, in polarized epithelial cells, such as those in renal epithelia, the process of primary cilium biogenesis takes place by an alternative route that occurs entirely at the cell surface^11,12^. In these cells, the midbody, which is an amorphous electron-dense structure situated in the middle of the intercellular bridge during cytokinesis, is inherited as a remnant and transits along the apical surface to meet the centrosome, where it licenses it for primary cilium assembly^13^. Ciliary length is controlled by multiple proteins and mechanisms^14,15^. Membrane trafficking machinery, such as annexin 13, syntaxin 3, the exocyst complex and Rab-family GTPases control ciliary length, probably by transporting ciliary materials to the centrosome region^16–19^. Recent studies have shown that the MAL protein affects the size of primary cilia by regulating correct membrane condensation at the ciliary base, which is required for efficient cilium elongation^20^. The actin cytoskeleton regulates the size of cilia by modulating the vesicular trafficking to the centrosome^21–23^. The balance between the anterograde/retrograde intraflagellar transport machinery, protein kinases^24^, cell signaling proteins and tubulin posttranslational modifications^25^ also contribute to the regulation of ciliary length.

Caveolin-1 (Cav1) is a membrane protein expressed as two isoforms, Cav1α and Cav1β, which arise from activity at two alternative translation initiation sites^26^. Cav1 is mainly known as a component of small, flask-shaped invaginated domains (caveolae), but is also present in non-caveolar flat membrane domains whose functions are still being investigated^27^. A broad variety of growth factor receptors, signaling kinases and other signaling molecules have been localized to Cav1 domains^27–29^. Although Cav1 domains and primary cilia are known to be important signaling hubs, the communication between them has not yet been thoroughly explored.

In this study, we have investigated the mechanism by which Cav1 modulates the length of the cilium. We analyzed the effect of knocking-down (KD) Cav1 in different cell lines that relied on distinct routes of primary cilium formation and observed that they all had longer cilia than control cells. The mechanism underlying this effect was further studied in Madin-Darby canine kidney (MDCK) epithelial cells. Knockout (KO) of Cav1 MDCK cells corroborated the effect of Cav1 KD. Remarkably, Cav1α exogenously expressed in Cav1 KO cells localized at the apical membrane and restored normal cilium length. Consistent with the importance of Cav1α, KO of only Cav1α also yielded long cilia. In addition, we found that Cav1α modulates Rho GTPase activity, which in turn, controls the apical actin meshwork through its effectors, ROCK and DIA1, to allow the delivery of extra material for primary cilium assembly. Since MDCK cells do not have caveolae at the apical membrane^30^, we propose that flat non-caveolar apical Cav1 domains, also known as Cav1 scaffolds, control cilium size by regulating RhoA activity.

## Results

### Cav1 knockdown affects primary cilium length

**Figure 1:**
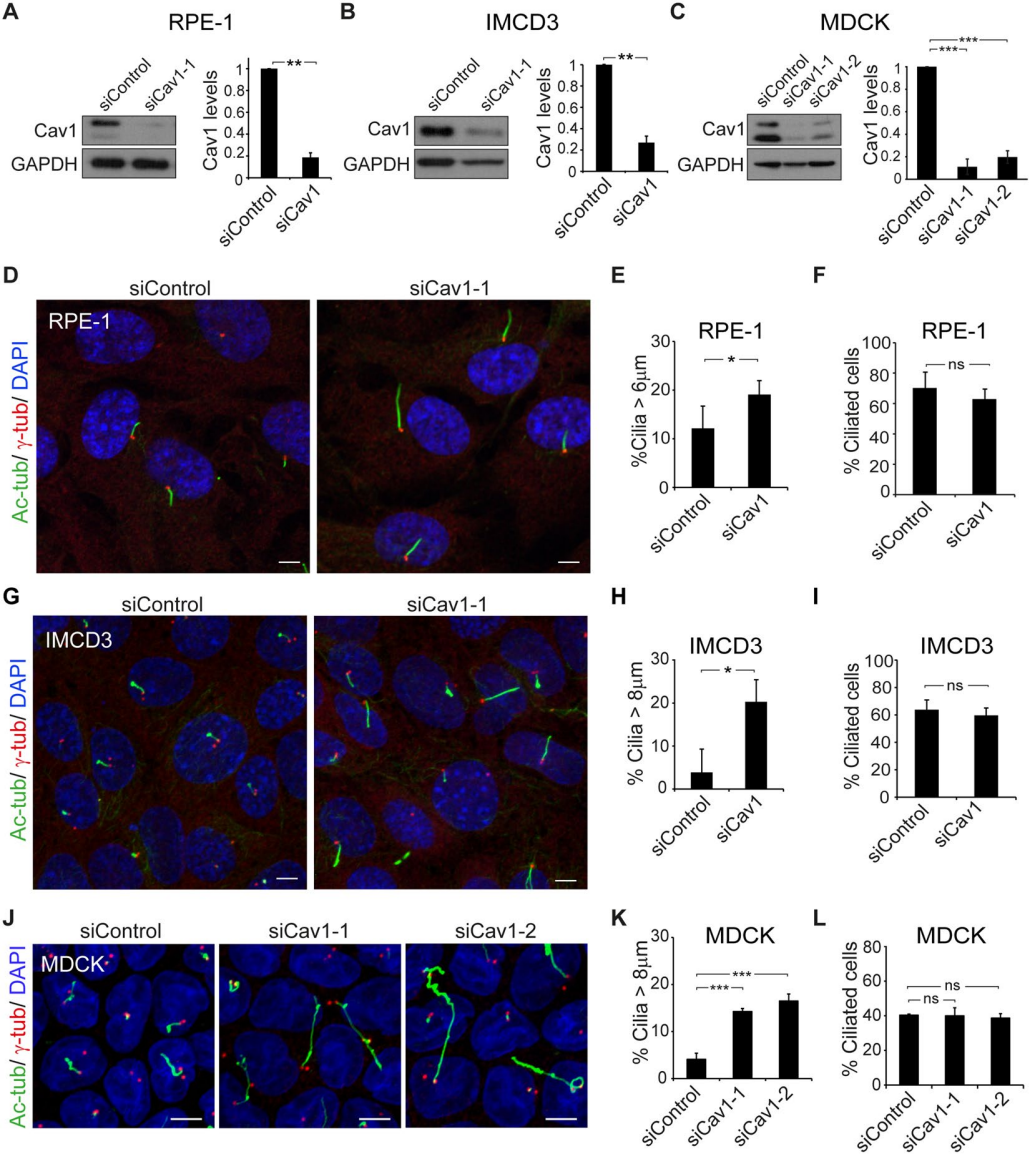
Cav1 knockdown induces cilium lengthening. (**A–C)** RPE-1, IMCD3 and MDCK cells were transfected with control siRNA or siRNAs targeting Cav1, and immunoblotted for Cav1. GAPDH was used as a protein-loading control (left panels). Histograms showing the expression levels of Cav1 in cells transfected with the indicated siRNAs relative to control siRNA. Cav1 signals were normalized to GAPDH (right panels). **(D**–**L)** RPE-1 (**D**) and IMCD3 (**G**) cells were grown on coverslips for 48 h and serum-starved for 24 h and MDCK (**J**) cells were grown on Transwell inserts for 96 h. All cell lines were then stained for acetylated tubulin (Ac-tub), γ-tubulin (γ-tub) and nuclei (DAPI). (**E**,**H**,**K**) Histograms illustrating the percentage of cilia longer than 6 µm (**E**) and 8 µm (H, K) for each type of cell. (**F**,**I**,**L**) Histograms representing the percentage of ciliated control and Cav1 KD cells. Scale bars, 5 µm. Data were pooled from three independent experiments and are presented as the mean ± SD. *P < 0.05; **P < 0.01; ***P < 0.001; ns, non-significant.

To investigate whether Cav1 regulates ciliary length independently of the cell type and the route by which primary cilium biogenesis occurs, we analyzed human retinal pigment 1 (RPE-1) cells, mouse inner medullary collecting duct 3 (IMCD3) cells, and canine renal MDCK cells. The primary cilium of RPE-1 cells is present in a deep invagination of the plasma membrane, known as the ciliary pocket, whereas that of MDCK cells protrudes directly from the cell surface. The presence or absence of the ciliary pocket appears to be the consequence of the use of the intracellular or alternative route of primary cilium assembly, respectively^12,31^. In the case of IMCD3 cells, approximately 90% of the ciliated cells have no pocket and use the alternative route, whereas the others rely on the intracellular pathway. To investigate the requirement of Cav1 for correct ciliogenesis, we first used a loss-of-function strategy based on small interference RNAs (siRNAs). The expression of specific siRNAs considerably reduced Cav1 levels in the three cell lines (Fig. 1A–C). Cells were stained for acetylated tubulin and γ-tubulin to visualize the primary cilium and the centrosome, respectively. Cav1 silencing did not alter the number of ciliated cells but did notably increase the size of the cilia in all cases (Fig. 1D–L). These results indicate that Cav1 modulates ciliary length regardless of the route used for primary cilium biogenesis.

### The Cav1α isoform is necessary for normal primary cilium length

**Figure 2:**
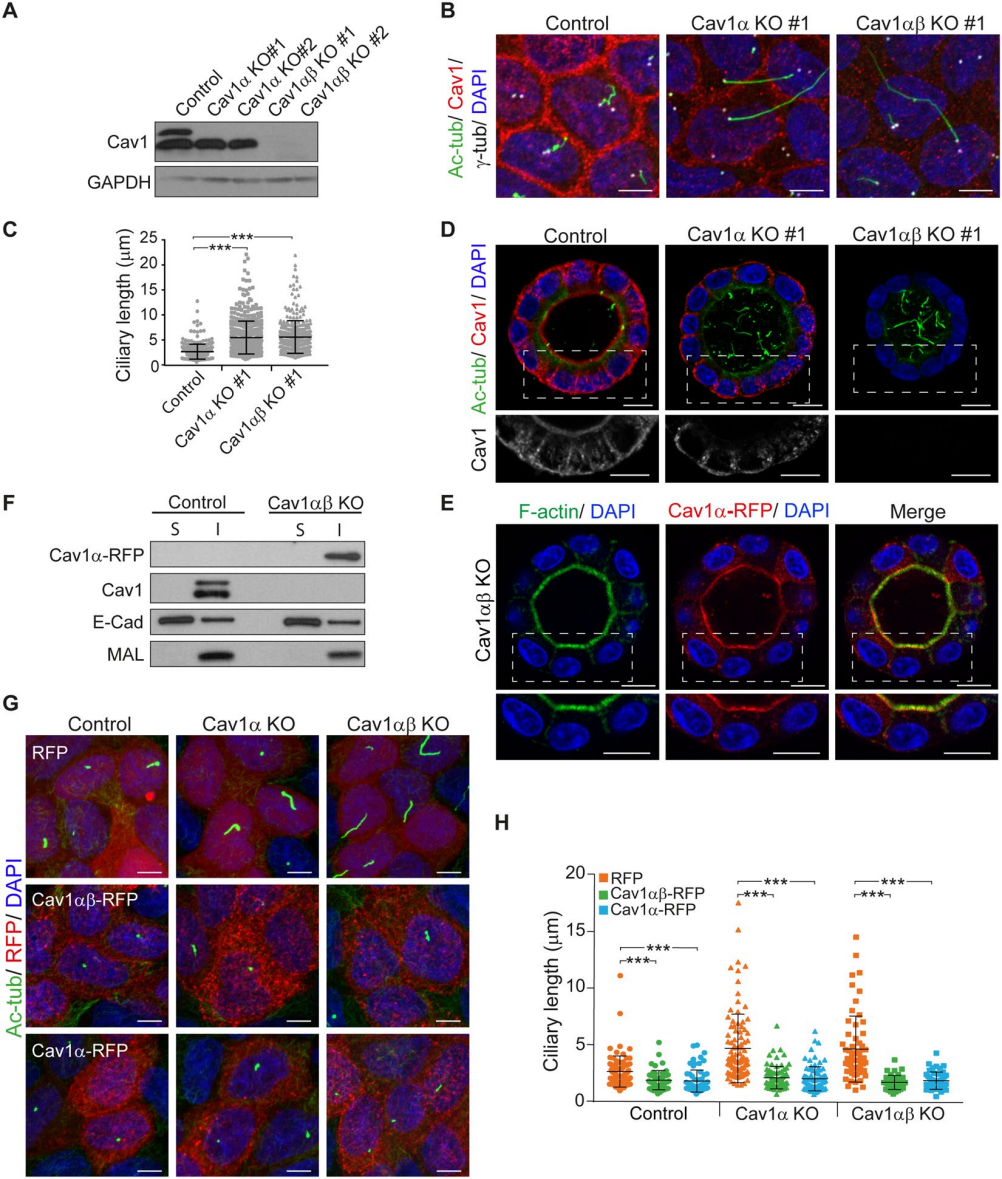
The isoform Cavα1 is responsible for primary cilium lengthening. (**A)** Control, Cav1α KO and Cav1αβ KO MDCK cells were analyzed by immunoblotting for Cav1. GAPDH was used as a protein-loading control. **(B)** Cells were cultured on Transwell inserts for 5 days, and then stained for acetylated tubulin, Cav1, γ-tubulin and nuclei. **(C)** The scatter-plot represents cilium lengths measured in µm; more than 700 cells were analyzed for each condition. **(D)** Cells cultured in 3D for 5 days were stained for acetylated tubulin, Cav1 and nuclei. The boxed regions are enlarged in the bottom panels to show solely Cav1 staining. **(E)** Cav1αβ KO cells were transiently transfected with Cav1α-RFP, cultured in 3D for 4 days, and stained for F-actin and nuclei. The boxed region of the cyst is shown enlarged in the lower panels. **(F)** Cav1αβ KO cells expressing Cav1α-RFP and untransfected control cells were cultured for 2 days. The soluble fraction (S) and the detergent-insoluble membrane fraction (I) were immunoblotted for Cav1. The distributions of E-cadherin and MAL were used as markers of the S and I fractions, respectively. **(G)** Control, Cav1α KO and Cav1αβ KO cells were transiently transfected with the indicated constructs, fixed at 4 days post-transfection and stained for acetylated tubulin and nuclei. **(H)** The scatter-plot represents cilia lengths measured in µm; more than 200 cells were evaluated in each case. Scale bars, 5 µm (**B**,**G**); 10 µm (**D**,**E**). Data were pooled from three independent experiments and presented as means ± SD. ***P < 0.001.

To investigate in more detail the effect of Cav1 depletion on primary cilium length, we chose MDCK cells since they are a paradigm of polarized epithelial cells^32^, assemble a primary cilium that projects perpendicularly from the apical surface, thereby facilitating its analysis by confocal microscopy, and present longer cilia than RPE-1 and IMDC3 cells. The two Cav1 isoforms differ in 31 amino acids, which are N-terminal in the Cav1α but absent from Cav1β^26^. We used CRISPR/Cas9 gene editing technology with an sgRNA targeting sequence located between the first and second translation initiation sites to generate stable Cav1α knockout cells (Cav1α KO cells), and a second sgRNA targeting sequence downstream of the second translation initiation site to generate stable KO cells for both Cav1α and Cav1β (Cav1αβ KO cells). Immunoblot analysis of different clones confirmed that Cav1α KO cells expressed only the β isoform and that neither isoform was present in Cav1αβ KO cell clones (Fig. 2A). Cells were grown for 5 days on Transwells to allow primary cilium formation and were analyzed by confocal microscopy. Cav1α KO clones lacked Cav1 staining at the apical membrane but not at the basolateral membrane, indicating Cav1β distribution at those sites. Cav1αβ KO cells presented only background signal (Figs 2B and S1A). Analysis of Cav1 αβ KO showed significantly longer cilia than those of control cells, confirming the results obtained with Cav1 siRNA. It is of note that silencing only Cav1α reproduced the results obtained in Cav1αβ KO cells (Figs 2C and S1B). In conclusion, these results indicate that Cav1α expression is necessary for maintaining normal ciliary length.

MDCK cells grown embedded in a gel of extracellular matrix generate hollow spheres or cysts, recapitulating part of the process of epithelial tube morphogenesis^33^. Cysts formed by Cav1α KO cells and Cav1αβ KO cells assembled longer cilia than those of control cells (Figs 2D and S1C). This effect is consistent with that obtained in a siRNA-based screen of vesicular transport-related proteins^17^. In control cysts, Cav1 distributed in intracellular vesicles and at the apical and basolateral membranes. However, the apical distribution of Cav1 was lost in Cav1α KO cysts. This result, which is consistent with previous work on MDCK cells using antibodies to the N-terminal sequence specific to Cav1α^34^, suggests that Cav1α localizes at the apical membrane in polarized epithelial cells. Indeed, the apical membrane localization of Cav1α was confirmed by expressing Cav1α fused to red fluorescent protein (Cav1α-RFP) in Cav1αβ KO cells (Fig. 2E).

Cav1 is known to partition preferentially into detergent-insoluble membrane fractions, which are enriched in condensed membranes^35,36^. To analyze the partition of Cav1α in the absence of Cav1β, we expressed Cav1α in Cav1αβ KO cells. We observed that, similar to total Cav1, Cav1α incorporated preferentially into the insoluble membrane fractions (Fig. 2F), indicating that, even in the absence of Cav1β, Cav1α associates with these specialized membranes. The results illustrated in Fig. 2E,F suggest that Cav1α is present in condensed membrane domains at the apical membrane regardless of the expression of Cav1β.

To confirm the role of Cav1α in the control of ciliary length, we expressed Cav1α-RFP or Cav1αβ-RFP and measured the size of the cilia 4 days post-transfection. Cav1αβ-RFP expression in Cav1α KO and Cav1αβ KO cells reduced the length of cilia to values similar to that of control cells transfected with only RFP (Fig. 2G,H). Cav1α-RFP expression in Cav1α KO cells led to cilia of similar size to those in control cells and, remarkably, those in Cav1αβ KO cells (Fig. 2G,H). These results confirm that Cav1α is necessary and sufficient to regulate the size of the cilia.

### Ultrastructural analysis of primary cilia in Cav1α KO cells

**Figure 3:**
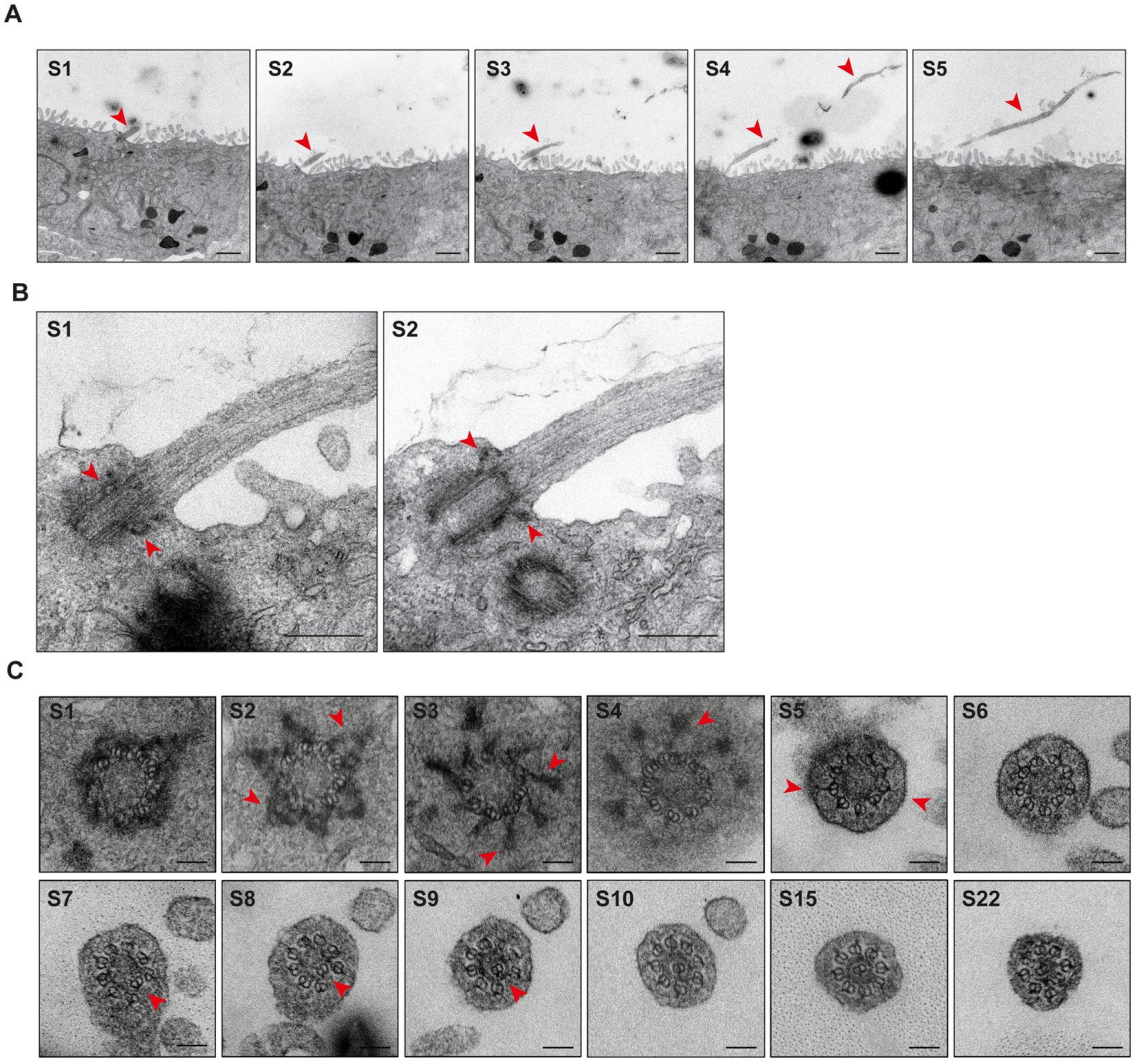
Ultrastructural analysis of a long primary cilium of Cav1α KO cells. MDCK cells were grown on Transwell inserts for 5 days and then fixed, embedded in resin and sectioned orthogonally or in parallel with the supporting substrate. **(A)** Representative TEM images of a longitudinally sectioned primary cilium (arrowhead) of Cav1α KO cells. Attention is drawn to the extreme length and curvature of the cilia in these cells. Serial sections (S1 to S5) are numbered from front to back. **(B)** Enlargements of representative sequential sections (S1 and S2) of a primary cilium of Cav1α KO cells. Arrowheads in S1 point to the basal feet, and in S2 indicate the transition fibers. **(C)** Representative TEM images of cross-sections of a primary cilium of Cav1α KO cells. Serial sections are numbered from the basal body (S1 to S4) to approximately 1.8 µm along the cilium (S5 to S22). Arrowheads indicate the basal feet in S2, the transition fibers in S3 and S4, Y-links in S5, and the microtubule doublet that moves to the center of the axoneme (S7 to S9). Note that, once in the central position, this doublet is surrounded by an electron-dense ring and the other eight microtubule doublets reorganize (S9 to S22). Scale bars, 1 µm (A); 0.4 µm (B); 0.1 µm (C).

We next investigated whether the ultrastructure of the long cilia of Cav1α KO is altered in any way. Consistent with our confocal microscopic observations, transmission electron microscopic (TEM) analysis of sequential XZ sections showed long cilia (Fig. 3A) with correctly positioned basal bodies at the plasma membrane (Figs 3B and S2A). Analysis of sequential cross-sections at the basal body level revealed normal microtubule triplets, basal feet and transition fibers (Figs 3C and S2B). The transition zone, which is above the basal body, presents characteristic Y-links of normal appearance. The 9 + 0 microtubule doublet pattern of the most proximal region of the axoneme was also normal. The pattern changed a little further along the cilium as one of the microtubule doublets gradually became displaced from the periphery and moved towards the center of the axoneme to become enclosed by an electron-dense ring. This effect was more apparent as the distance from the basal body increased. In parallel, the other doublets reorganized to fill the gap left by the migrated doublet, and the axoneme displayed an 8 + 1 doublet pattern (Figs 3C and S2B). This pattern has been previously observed in primary cilia of several cell types^37–39^. In summary, the results in Figs 3 and S2 indicate that the long cilia of Cav1α KO cells have a normal ultrastructure.

### Cav1α controls the apical actin cytoskeletal meshwork

**Figure 4:**
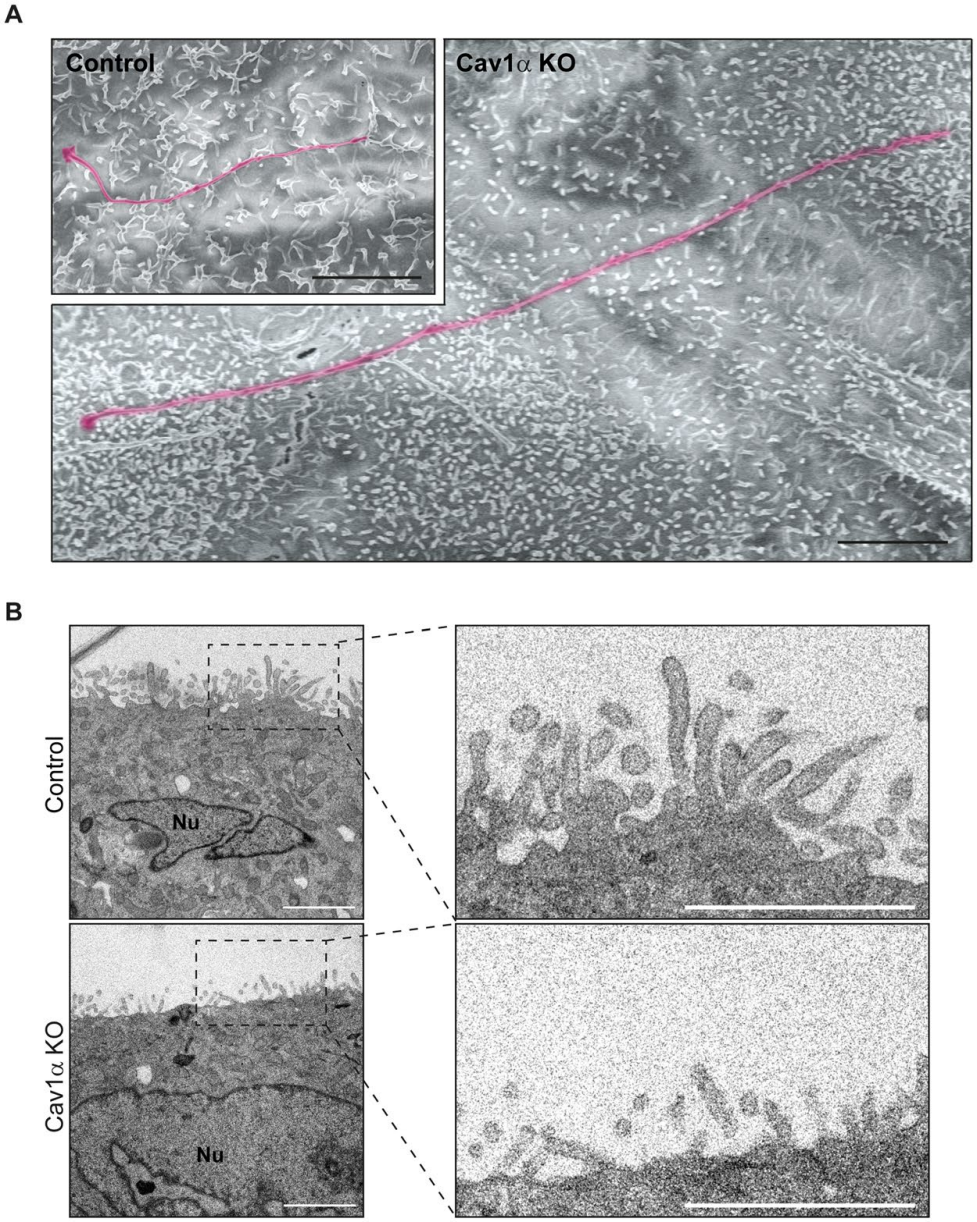
Effect of Cav1α knockout on microvilli length. Control and Cav1α KO cells were grown on Transwell inserts for 5 days, then fixed and treated for (A) SEM and (B) TEM analysis. (**A**) Representative SEM images showing an extremely long primary cilium of Cav1α KO cells compared with a representative cilium of control cells (magenta color). Cav1α KO cells present considerably shorter apical microvilli than control cells. The two images are at the same scale. (**B**) Representative XZ TEM images confirm that Cav1α KO cells have shorter microvilli than control cells. The right panels show enlargements of the boxed regions. Nuclei (Nu) are indicated. Scale bars, 3 µm (A); 2 µm (B).

Since the sole KO of Cav1α produced long cilia and Cav1α distributes apically (Fig. 2B,D,E), we examined the existence of morphological alterations in the apical surface of Cav1α KO cells by scanning electron microscopy (SEM). We observed that the overall morphology of the long cilia of Cav1α KO cells did not differ from that of cilia in control cells (Fig. 4A). However, it is of particular note that the microvilli of Cav1α KO cells were significantly shorter than those of control cells. The presence of abnormally short microvilli was confirmed by TEM (Fig. 4B). Since microvilli are actin-based protrusions, the results in Fig. 4 suggest that the absence of Cav1α expression alters the apical actin meshwork.

**Figure 5:**
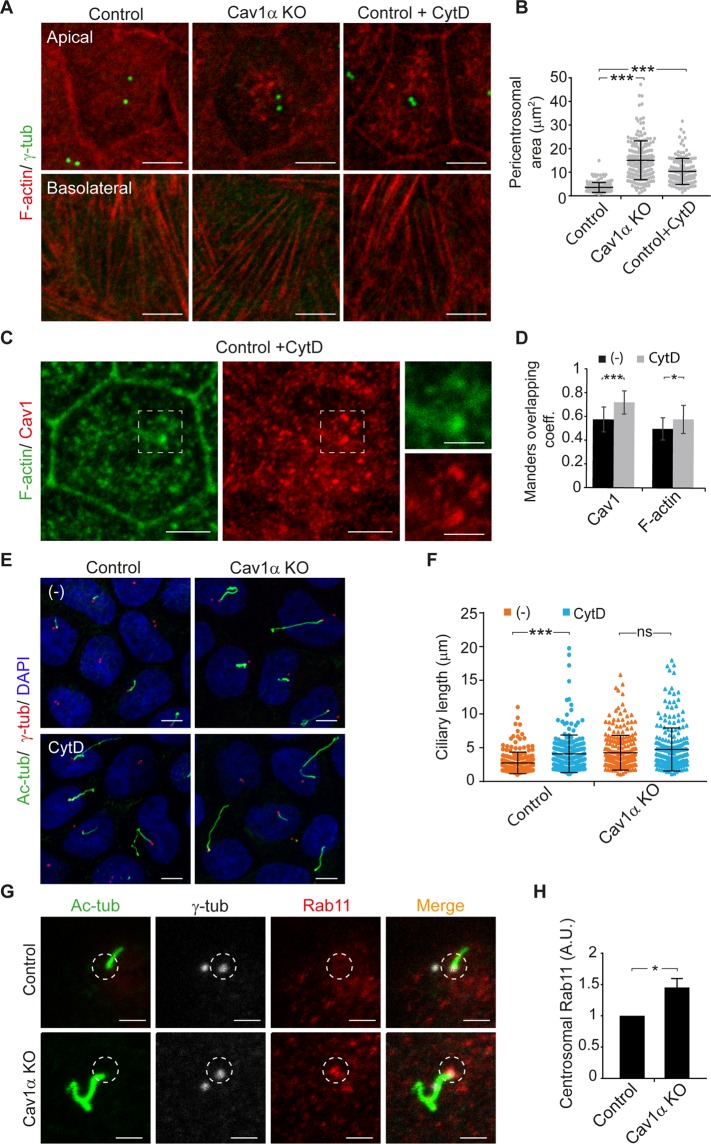
Cav1α knockout impairs apical actin network that leads to cilium lengthening. **(A–F)** Cells were grown on Transwells for 5 days, then fixed and analyzed by confocal microscopy. (**A**) Immunofluorescence images showing the apical (top panels) and basolateral (bottom panels) membrane of control and Cav1α KO cells and of control cells treated with 100 nM cytochalasin D (CytD) for 5 h and stained for F-actin and γ-tubulin. (**B**) The scatter-plot represents the area of the pericentrosomal region that lacks F-actin staining. (**C**) Immunofluorescence images showing the apical membrane of control cells treated with 100 nM CytD for 5 h stained for F-actin and Cav1. The boxed regions are shown enlarged at the right; scale bars, 2 µm. (**D**) The histogram shows the Mander’s overlap coefficient between Cav1 and F-actin in untreated control cells and in cells treated with CytD. (**E**) Immunofluorescence images of control and Cav1α KO cells untreated (−) or treated with CytD stained for acetylated tubulin, γ-tubulin and nuclei. (**F**) The scatter-plot represents cilium lengths measured in µm; more than 650 cells were evaluated for each condition. **(G**,**H)** Control and Cav1α KO cells were grown for 3 days and stained for acetylated tubulin, γ-tubulin and Rab11. The circles indicate the selected region at the basal body for Rab11 intensity analysis (**G**). The histogram represents the mean fluorescence intensity of Rab11 at the centrosome zone in Cav1α KO cells relative to control cells; n = 90 cells per experimental point (**H**). Scale bars, 5 µm. Data in B, D, F and H were from three independent experiments and are presented as the means ± SD. *P < 0.05; ***P < 0.001; ns, non-significant.

MDCK cells cultured in Transwell filters acquire a columnar shape that facilitates the analysis of apical and basolateral sections by confocal microscopy. To examine the apical actin cytoskeleton in Cav1α KO cells, we grew control and Cav1α KO cells in Transwell filters and analyzed the distribution of filamentous actin (F-actin). Whereas F-actin in control cells had a diffuse distribution throughout the entire apical membrane and accumulated at the cell-cell junctions (Fig. 5A), it concentrated in apical patches in Cav1α KO cells. By contrast, the distribution of F-actin at cell-cell junctions and basal stress-fibers was not affected (Fig. 5A). Notably, the F-actin-free zone surrounding the centrosome was larger in Cav1α KO cells (∼15.1 µm^2^) than that in control cells (∼3.5 µm^2^) (Fig. 5B). It is of note that the expression of Cav1α in Cav1α KO cells recovered the F-actin distribution observed in control cells (Fig. S3A). These results indicate that Cav1α expression is important for the correct organization of the apical actin meshwork.

Treatment with cytochalasin D (CytD), which inhibits actin polymerization and thereby disrupts the actin cytoskeleton, produces long cilia^23^. Since we observed an abnormal apical actin meshwork in Cav1α KO cells, we compared it with that provoked by CytD treatment. Control cells grown in Transwell filters for 5 days were treated for 5 h with CytD under conditions that disrupted the apical actin cytoskeleton but did not affect the distribution of the more stable F-actin at cell-cell junctions and the basal stress fibers (Fig. 5A). Similarly to what occurs in Cav1α KO cells, apical F-actin appeared more clustered and there was less F-actin at the pericentrosomal zone in CytD-treated control cells compared with untreated cells (Fig. 5A,B). Notably, both apical Cav1 and actin distributed in the apical patches observed in CytD-treated control cells (Fig. 5C,D). Since CytD induces long cilia^23^, we wondered whether the long cilia observed in Cav1α KO cells were due to the defects observed in the apical actin meshwork. To address this question, control and Cav1α KO cells were treated or not with CytD and the size of their cilia was compared. Whereas CytD treatment induced a dramatic lengthening of cilia in control cells (∼4.1 µm in CytD-treated vs. ∼2.7 µm in untreated control cells; Fig. 5E,F), it did not induce a significant increase in cilia size in Cav1α KO cells (∼4.7 µm in CytD-treated vs. ∼4.3 µm in untreated Cav1α KO cells; Fig. 5E,F). The similarity in the size of cilia between control cells treated with CytD and those of untreated Cav1α KO cells, in conjunction with the lack of effect of CytD on the size of cilia of Cav1α KO cells, is consistent with the possibility that the long cilia of Cav1α KO cells are due to defects in the apical actin meshwork.

It has been proposed that the long cilia observed in CytD-treated cells are caused by F-actin-clearing at the ciliary base, which allows more transport vesicles to arrive, thereby providing the extra material for ciliary growth^23^. To determine whether this could also occur in Cav1α KO cells, we analyzed Rab11 vesicles at the centrosome zone in control and Cav1α KO cells. We found an increased number of Rab11 vesicles in the absence of Cav1α expression (Fig. 5G,H).

Measurements of ciliary length at different times after cell seeding indicates that initially (after 24 h) the length was similar in control and Cav1 KO cells. At later times, Cav1 KO cells had longer cilia than those in control cells, this difference increasing progressively (Fig. S3B). This observation indicates that there is no apparent difference in the initial rate of cilium assembly between the two types of cell, despite the presence of extra material in the pericentrosomal zone of Cav1 KO cells. However, once the cilium has formed, the excess material causes the primary cilium to become oversized. In conclusion, it seems that Cav1α regulates the apical actin meshwork, which, in turn, modulates the arrival of more ciliary precursors at the centrosome that can manufacture long cilia.

### Cav1α controls cilium length by modulating Rho GTPase activity

**Figure 6:**
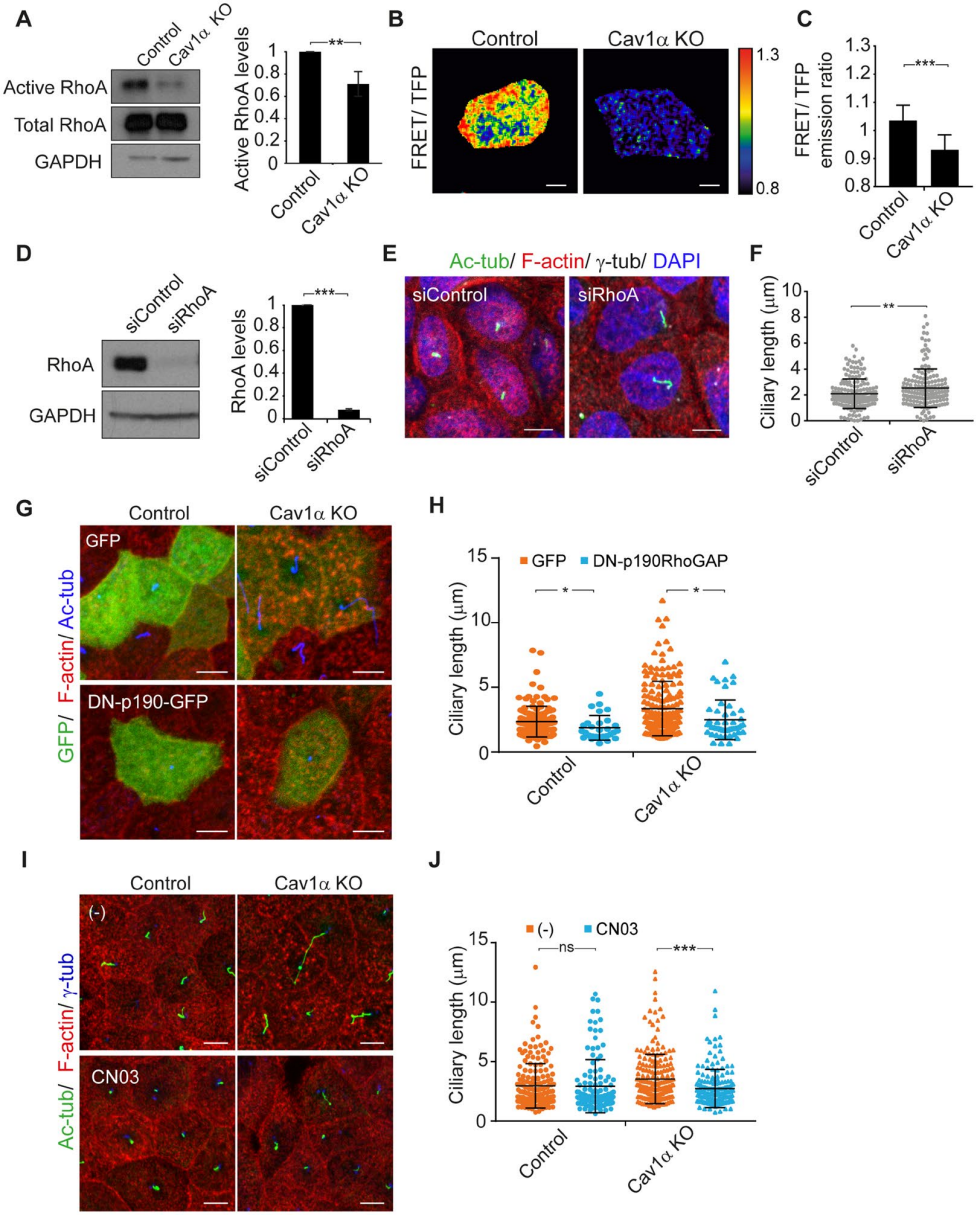
Cav1α knockout reduces RhoA activity. **(A)** GTP-bound RhoA from control and Cav1α KO cells were pulled-down by GST-rhotekin-RBD and subjected to immunoblot analysis for total and active RhoA levels (left panel). The histogram represents active RhoA normalized to total RhoA levels in Cav1α KO cells relative to control cells (right panel). **(B,C)** FRET-based analysis of RhoA activity was done in living control and Cav1α KO cells, grown for 5 days. FRET efficiency is shown on the right with the upper and lower limits of the ratio range indicated in the color bar (**B**). The histogram represents the quantitative measurements of FRET/TFP ratios in the apical membrane; n = 50 cells per condition (**C**). **(D)** Control cells were treated with control siRNA or siRNA targeting RhoA. Cell extracts were immunoblotted for RhoA, and GAPDH was used as a control of protein loading (left panels). The histogram shows the expression levels of RhoA in cells transfected with the indicated siRNAs. RhoA signals were normalized to GAPDH (right panel). **(E,F)** Control and RhoA KD cells were grown for 72 h after transfection and stained for acetylated tubulin, F-actin, γ-tubulin and nuclei (**E**). The scatter-plot represents ciliary lengths, measured in µm, in cells transfected with the indicated siRNAs; more than 575 cells were analyzed for each condition (**F**). **(G,H)** Control and Cav1α KO cells were transfected with GFP or DN-p190RhoGAP-GFP for 3 days and then analyzed by immunofluorescence. Cells were stained for F-actin and acetylated tubulin (**G**). The scatter-plot represents the length of cilia measured in µm (**H**). **(I,J)** Control and Cav1α KO cells were untreated (−) or treated with 0.5 µg/ml CN03 for 4 h and then analyzed by immunofluorescence. Cells were stained for acetylated tubulin, F-actin and γ-tubulin (**I**). The scatter-plot represents the length of cilia measured in µm; more than 250 cells were analyzed for each condition (**J**). Scale bars, 5 µm. Data were pooled from three independent experiments and are presented as the mean ± SD. *P < 0.05; **P < 0.01; ***P < 0.001; ns, non-significant.

The small GTPase Rho family of proteins and their effectors orchestrate the organization of the actin cytoskeleton^40^. Among them, RhoA plays an important role in regulating the apical cytoskeleton in polarized epithelial cells^41,42^. To assess whether the disruption of the apical actin meshwork observed in Cav1α KO cells is caused by RhoA regulation, we carried out pull-down assays to measure RhoA activity. Immunoblot analysis showed that total RhoA levels were similar in control and Cav1α KO cells. By contrast, RhoA activity was lower in Cav1α KO cells (Fig. 6A). To confirm this result by an independent technique, we used the fluorescence resonance energy transfer (FRET) biosensor Raichu-RhoA^43^. Only cells with a very low level of biosensor expression were analyzed to avoid conflating these effects with others due to overexpression. Consistent with the pull-down assays, FRET analysis showed that RhoA activity at the apical membrane was lower in Cav1α KO cells than in control cells (Fig. 6B,C). Therefore, our results indicate that Cav1α modulates RhoA activity at the apical membrane.

To determine whether RhoA expression affects ciliary length, we analyzed RhoA KD cells (Fig. 6D) and found their cilia to be longer than those of control cells (Fig. 6E,F). Consistent with this finding, the expression of a dominant-negative mutant of p190RhoGAP^44^, which inhibits Rho GTPase activity and increases the levels of active GTP-loaded Rho, reduced the length of cilia in control and Cav1α KO cells (Fig. 6G,H). To confirm the involvement of Rho in the control of cilia length by Cav1α, we used the Rho activator CN03. Rho activation completely restored normal cilium length in Cav1α KO cells (∼2.7 µm in CN03-treated Cav1α KO cells versus ∼2.9 µm in untreated control cells; Fig. 6I,J). The alteration of the subapical actin meshwork in RhoA KD cells was similar to that observed in Cav1α KO cells (Figs 6E and S4A). RhoA activation in Cav1α KO cells by the expression of a dominant-negative p190RhoGAP or by CN03 treatment restored subapical actin distribution (Figs 6G,I and S4B,C). In conclusion, Cav1α appears to control cilium length by modulating Rho activity, which in turn, regulates the apical actin meshwork.

### Cav1α controls cilium length via ROCK and DIA1

**Figure 7:**
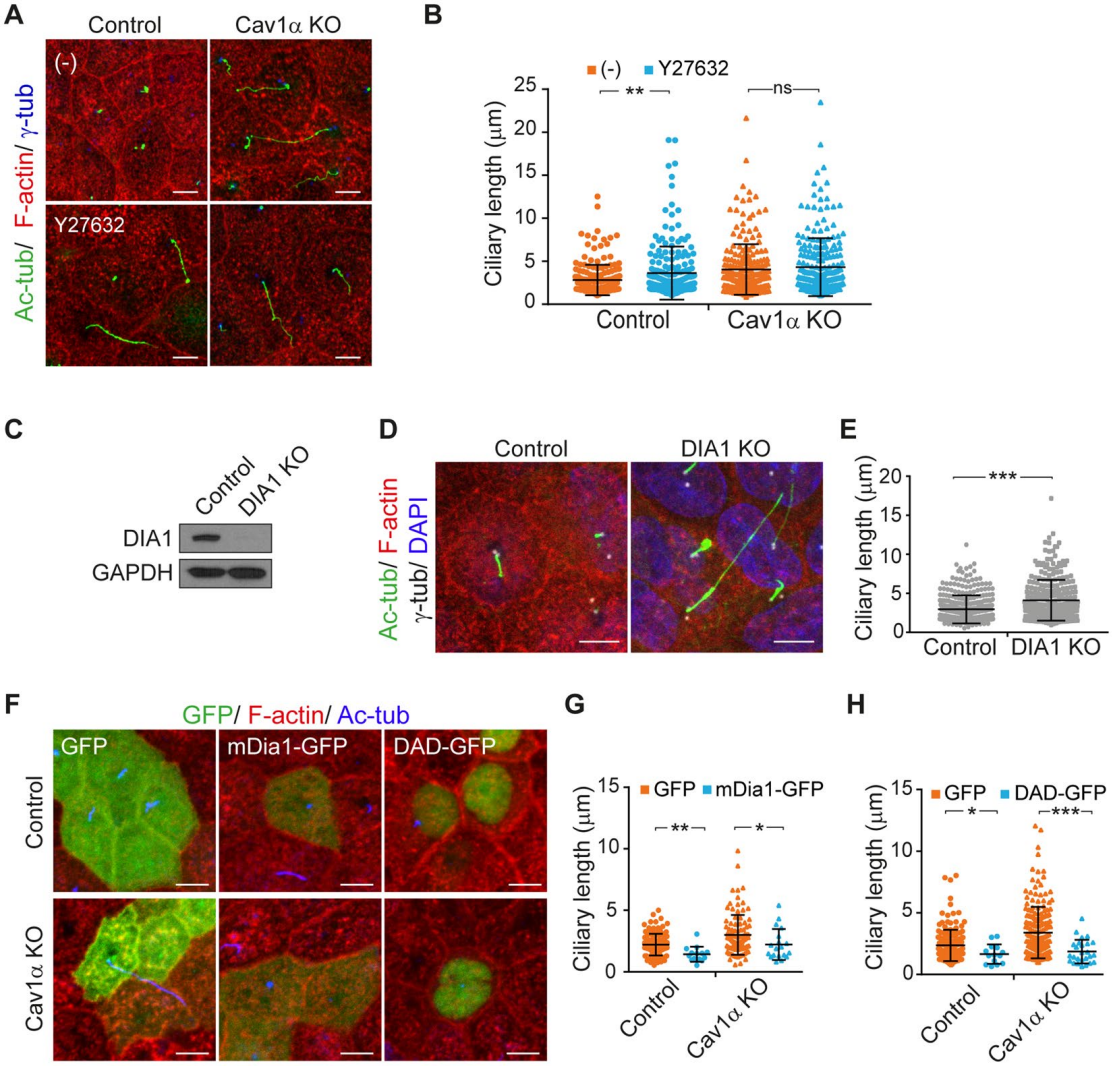
Cav1α controls cilium length via ROCK and Dia1. **(A**,**B)** Control and Cav1α KO cells were untreated (−) or treated with 10 µM Y27632 for 24 h and then stained for acetylated tubulin, F-actin and γ-tubulin (**A**). The scatter-plot represents the total length of cilia measured in µm; more than 700 cells were analyzed for each condition (**B**). **(C)** Control and DIA1 KO cells were analyzed by immunoblotting for DIA1. GAPDH was used as a loading control. **(D**,**E)** Control and DIA1 KO cells were grown for 5 days and stained for acetylated tubulin, F-actin, γ-tubulin and nuclei (**D**). The scatter-plot represents ciliary lengths measured in µm; more than 700 cells were analyzed in control and DIA1 KO cells (**E**). **(F–H)** Control and Cav1α KO cells were transiently transfected with the indicated constructs, then fixed and stained after 72 h for F-actin and acetylated tubulin (**F**). The scatter-plots represent the ciliary lengths measured in µm; more than 100 cells were analyzed for each condition (**G**,**H**). Scale bars, 5 µm. Data in B, E, G and H were pooled from at least three independent experiments and are represented as the mean ± SD. *P < 0.05; **P < 0.01; ***P < 0.001; ns, non-significant.

RhoA regulates the actin cytoskeleton through its effectors, Rho-associated kinase (ROCK) and the formin mDia^45^. To gain insights into the regulation of ciliary size via RhoA signaling, we used the ROCK inhibitor Y-27632^46^. Control cells treated with Y-27632 showed disrupted apical cytoskeleton and, consistent with the involvement of ROCK in the control of ciliary length, longer cilia than untreated cells (∼3.6 µm in Y-27632 treated cells compared with ∼2.8 µm in untreated control cells), reaching a mean length close to that in Cav1α KO cells (∼4.1 µm; Figs 7A,B and S4D). Since ROCK inhibition did not cause any further increase of cilium length in Cav1α KO cells (Fig. 7A,B), we may conclude that ROCK and Cav1α regulate cilium length through the same pathway.

The mDia1 molecule closes and acquires an inactive conformation when the diaphanous autoregulatory domain (DAD) interacts with the diaphanous inhibitory domain (DID), which are present at the carboxyl and amino terminus, respectively. The binding of GTP-loaded RhoA to the DID promotes the release of mDia1 from its autoinhibitory conformation, allowing actin nucleation and polymerization^47,48^. To evaluate whether endogenous DIA1, which is the canine counterpart of mDia1, helps regulate ciliary length, we generated DIA1 KO MDCK cells (Fig. 7C). The apical actin cytoskeleton was disrupted in DIA1 KO cells and cilia were significantly longer than those of control cells (∼4.1 µm vs. ∼2.9 µm; Figs 7D,E and S4E). The requirement of DIA1 for normal cilium size was specific since cells KO for the formin INF2, which is not regulated by RhoA, had no effect (Fig. S5A–C). To confirm a role for DIA1 in the regulation of ciliary size, we overexpressed mDia1-GFP in Cav1α KO cells and found that it restored normal ciliary length and apical actin organization (∼2.2 µm in mDia-GFP transfected cells compared with ∼3.1 µm in GFP-transfected cells). Consistent with a role for DIA1, we also detected a reduction in the size of the cilia in control cells expressing mDia1-GFP (∼1.4 µm in mDia-GFP transfected cells compared with ∼2.2 µm in GFP-transfected cells), suggesting that an excess of F-actin impedes normal cilium growth (Figs 7F,G and S4F). Similar results were obtained from the exogenous expression of the DAD of DIA1 (Figs 7F,H and S4F), which activates endogenous DIA1 by disrupting the intramolecular DID-DAD interaction^49^. As a control, we observed that expression of mDia1-GFP in DIA1 KO cells rescued cilium length (Fig. S5D,E), but expression of mDia1 DAD did not (Fig. S5D,F). The results presented in Figs 7 and S4 suggest that Cav1α controls cilium length through both ROCK and DIA1.

It has been suggested that mDia1 and ROCK work concurrently during Rho-induced actin reorganization^50,51^. Given that both proteins regulate ciliary lengthening, we evaluated their relative contributions. We observed that inhibition of ROCK in DIA1 KO cells provoked a further increase in the size of cilia (∼4.0 µm vs. ∼3.6 µm in untreated DIA1 KO cells; Fig. S6A,B). This result, together with the finding that the effect of ROCK inhibition on the increase in cilium size was greater in control cells, indicates that ROCK and DIA1 act together to regulate ciliary length.

Collectively, the results presented in this study show that Cav1α acts via RhoA and its effectors, ROCK and DIA1, to regulate the apical actin meshwork, which affects the arrival of vesicles to the ciliary base, which subsequently affects cilium length.

## Discussion

The variation in the size of the cilium among distinct cell types suggests that primary cilium length is subject to biological regulation. In principle, its length is regulated by the availability of materials at the centrosome to make up the ciliary membrane, the balanced equilibrium between the anterograde and retrograde activity of the intraflagellar transport machinery, and mechanisms of ciliary membrane removal^14,15^. In this study, we have investigated the mechanism by which Cav1 regulates primary cilium length. We found that Cav1α expression regulates the arrangement of the apical actin meshwork of epithelial MDCK cells. Cav1α does so by a mechanism involving the GTPase RhoA and its effectors, ROCK and DIA1. In the absence of Cav1α, the apical actin meshwork is disrupted and more vesicles feed the centrosome for ciliary membrane assembly and, as result, cilia became abnormally long. The presence of Cav1α at the apical membrane was found to be necessary and sufficient for the regulation of primary cilium size. As in MDCK cells, Cav1-silencing in RPE-1 and IMCD3 cells also produced long cilia. Therefore, we conclude that Cav1 has a general role in the regulation of cilium length.

Rho GTPases are key regulators of cytoskeletal dynamics and affect a large number of cellular processes, such as cell polarity, migration, vesicle trafficking and cytokinesis. Cav1 regulates Rho activity by at least two different mechanisms. In Cav1-deficient mouse embryonic fibroblasts, Src tyrosine kinase activity is constitutively high and modulates the p190RhoGAP-dependent decrease of GTP-loaded Rho levels, which subsequently remodels the actin cytoskeleton^52^. A second pathway involves the regulation of the GEF Vav-2, which associates with Cav1 and activates Cav1-bound Rho^53^. Consistent with the role of Cav1 in Rho regulation and the presence of Cav1α at the apical surface, Cav1α KO MDCK cells showed low RhoA activity and a disrupted apical actin meshwork.

The effect of Cav1α KO on cilium size appears to be due to disruption of the apical actin meshwork, since it is mimicked by treating control cells with suboptimal doses of CytD, which clears actin at the basal body, facilitating the arrival of transport vesicles^23^. Similarly, we found less actin at the centrosome and the centrosome fed with extra vesicles in Cav1α KO cells compared with control cells. Providing further evidence of the involvement of Rho downstream of Cav1α, we found that RhoA depletion in control cells produced long cilia, whereas Rho activation by treatment with CN03 or expression of dominant-negative p190RhoGAP reversed the effect of Cav1α KO on cilium size. In addition to long cilia, Cav1α KO cells exhibited short microvilli, similar to those reported in cells treated with CytD^54^. Unlike the microvilli of control cells, those of Cav1α KO cells were less clustered, which is consistent with reports showing that microvilli development is dependent on the Rho family of GTPases^55^. The observation that Cav1α KO cells presented a disrupted apical actin meshwork and abnormally long cilia, and that the sole expression of Cav1α corrected both defects indicates a specific function of Cav1α in the Rho-mediated organization of the apical actin cytoskeleton and, as a consequence, of cilium size regulation.

The Rho GTPase regulates the actin cytoskeleton mainly through its effectors, mDia and ROCK. mDia catalyzes actin nucleation and polymerization and produces long, straight actin filaments, whereas ROCK is a serine/threonine kinase that regulates downstream substrates of importance in actin cytoskeleton remodeling^56^. We found that DIA1 KO leads to increased ciliary size, while overexpression of mDia1 or activation of endogenous DIA1 reduces it. Similarly, ROCK inhibition increases ciliary length in control cells. It is of note that ROCK inhibition in DIA1 KO cells produces a further increase in the size of cilia. Therefore, both DIA1 and ROCK are important for regulating cilium size, and their individual contribution is necessary to ensure full control.

Cav1 partitions preferentially into detergent-insoluble membrane fractions, which are enriched in condensed membranes^35,36^. In MDCK cells, Cav1 is present in approximately equal amounts on the apical and basolateral surface^30^. At the basolateral membrane, most Cav1 is associated with caveolae. In contrast, apical Cav1 has not been reported in any discernible structure at the EM level^30^. Most studies of Cav1 associate its functions with caveolae, although increasing evidence indicates that Cav1 also contributes to cell regulation outside the caveolae^27^. After biosynthesis, Cav1 forms Cav1-Cav2 heterooligomers that are destined for the basolateral membrane and Cav1 homooligomers that are transported to the apical membrane. Cav1-Cav2 heterooligomers form caveolae, which are exclusive to the basolateral surface^30^. Cav1 homooligomers are thought to form flat, condensed membrane domains (5–100 nm), which are referred to as non-caveolar Cav1 domains, or Cav1 scaffolds, and which may exhibit many of the signaling regulatory proteins associated with Cav1^27^. We observed clustering of apical Cav1 structures in actin patches in CytD-treated control cells, suggesting that apical Cav1 could be organized in membrane domains. In addition, taking into account that: (i) no Cav1 staining was found at the apical membrane in Cav1α KO cells, (ii) exogenous Cav1α expressed in Cav-1αβ KO cells distributed exclusively at the apical surface, (iii) apical Cav1α partitions exclusively into detergent-insoluble membranes, and (iv) there are no caveolae at the apical membrane, it seems plausible that Cav1α is present at the apical membrane in non-caveolar domains. Caveolae control Rho activity and caveolae-linked actin cytoskeleton^57,58^. We propose that a similar control of Rho activity and the organization of the apical actin meshwork, which is required for cilium size regulation, operates in non-caveolar apical Cav1 domains.

Cav1 deficiency promotes autophagy^59–62^. Notably, the regulatory function of Cav1 in lysosome and autophagy seems to involve membrane rafts in a caveolae-independent manner^63^. There is a bidirectional crosstalk between primary cilium and autophagy. On the one hand, primary cilium signaling controls autophagosome formation while, on the other, autophagy regulates ciliogenesis and primary cilium length^64,65^. Therefore, although our results point to a role of Cav1-based scaffolds in controlling the length of primary cilia through modulation of the apical actin cytoskeleton, additional participation of autophagy in ciliary lenghtening cannot be ruled out.

Primary cilia are sensors of mechanical force. Assuming that rigidity is similar in all primary cilia, their length is intimately linked to the response to mechanical perturbation, for instance to fluid-flow stretching and compression^66^. Using cells cultured in adhesive micropatterns, it has been observed that the actin cytoskeleton is required for the response of primary cilia to cell confinement^67^. Although primary cilia are customarily considered to be non-motile, fluctuations in the actin-myosin network seem to cause cilia to move^68^. The distance swept by the tip of the cilium increases as the cilium lengthens and probably gives rise to different signaling intensities. Long cilia have recently been shown to amplify sonic hedgehog signaling^69^, and Cav1 was found to be important for establishing a microdomain at the ciliary membrane required for sonic hedgehog signaling^70^. It has been proposed that caveolae couple mechanotransduction pathways to actin-controlled changes in tension through their association with stress fibers^57^. The regulation of cilium length by Cav1α might be an adaptation of the apical surface, which lacks caveolae, to respond appropriately to mechanical perturbation. In addition, the capacity to control cilium length might serve to regulate the establishment of long-lasting contacts between distant cells^71^.

Cav1 KO mice were reported a long time ago^72,73^. Although many studies have been carried out on Cav1 KO mice, to our knowledge there are no reports analyzing primary cilia in these mice. Therefore, either cilia in Cav1 KO have not been studied, or they have normal cilia because the effect on cilia size we observed in tissue culture Cav1 KO cells is compensated in some way by other regulatory mechanisms in Cav1 KO mice. Some of the many abnormalities found in Cav1 KO mice (e.g., pulmonary defects, vascular dysfunction, obesity) have also been found in patients with ciliary dysfunction caused by the mutation of ciliary genes. Therefore, the possibility remains that abnormal cilia function may contribute to some of the disorders found in Cav1 KO mice.

**Figure 8:**
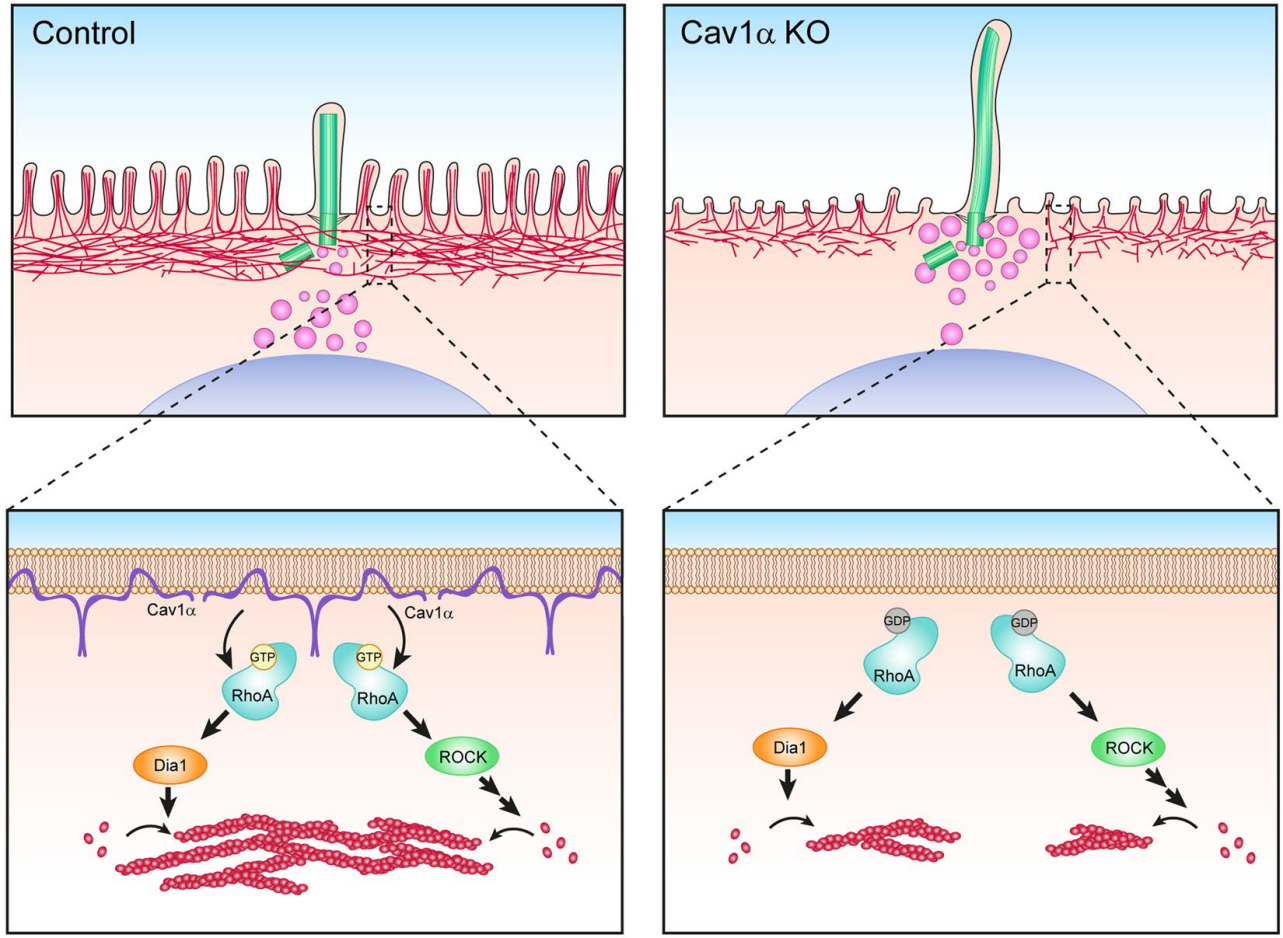
Model of Cav1α function in the regulation of primary cilia length. In control cells (left), Cav1α localizes in non-caveolar domains at the apical membrane of MDCK cells, where it positively regulates RhoA activity. Active RhoA subsequently promotes apical actin polymerization through its effectors ROCK1 and DIA1. Actin meshwork rearrangement regulates ciliary lengthening by controlling the access of transport vesicles to the centrosomal zone. In the absence of Cav1α (right), RhoA activation is impaired and less apical actin polymerizes, allowing the arrival of more material at the centrosome that is used to assemble longer cilia.

Cav1 domains and cilia orchestrate important signaling pathways. In this study, we have identified apical non-caveolar Cav1α as an important regulator of ciliary length and shown that Cav1α does this by modulating the activity of apical RhoA. In turn, RhoA regulates the organization of the apical actin cytoskeleton meshwork, through its effectors, DIA1 and ROCK, to allow the access of extra ciliary material to enable primary cilium lengthening (Fig. 8).

## Materials and Methods

### Antibodies and reagents

The sources of the antibodies to the different markers were as follows: Cav1 (mouse mAb IgG1, used at 1/2,000 for immunoblot analysis; 610406; and the rabbit polyclonal antibody used at 1/200 for immunofluorescence analysis; 610059) and DIA1 (mouse mAb IgG1, used at 1/1000 for immunoblotting; clone51/mDia1; 610484), were from BD Transduction Laboratories. γ-tubulin (mouse mAb IgG1, used at 1/500 for immunofluorescence analysis; clone GTU-88, T3559) and acetylated tubulin (mouse mAb IgG2b, used at 1/500 for immunofluorescence analysis; clone 6-11B-1; T7451) were obtained from Sigma-Aldrich. GAPDH (mouse mAb IgG1, used at 1/150,000 for immunoblotting; clone 6C5; AM4300) was from Ambion. RhoA (mouse mAb IgG1, used at 1/2,000 for immunoblotting; 26C4; sc-418) and MAL (goat polyclonal antibody, used at 1/500 for immunoblotting; T-18; sc-46171) were from Santa Cruz Biotechnology, Inc. E-Cadherin (mAb IgG1, used at 1/500 for immunoblotting; rr1) was from DSHB. The rabbit polyclonal antibody to INF2 (used at 1/500 for immunoblotting) has been previously described^74^. Cytochalasin D was from Sigma Aldrich. RhoA activator II (CN03) was from Cytoskeleton. ROCK inhibitor Y27632 was obtained from Calbiochem. Fluorescent phalloidin and secondary antibodies conjugated to Alexa Fluor-488, -555, -594 and -647 were from Life Technologies. The DAPI (268298) stain was from Merck. Horseradish peroxidase-labeled secondary antibodies were from Southern Biotechnology Associates and Jackson Immunoresearch Laboratories, Inc.

### Cell culture

Epithelial canine MDCK II cells (CRL2936) were grown in MEM supplemented with 5% fetal bovine serum (FBS). 5 × 10^5^ cells were seeded onto 12-mm polycarbonate membranes of 0.2 µm pore size (Costar Transwell, Corning), or 1 × 10^5^ cells on coverslips maintained in 24-multiwell plates. Cells were grown for the times indicated in each figure legend. Since the size of cilia increases with time, cilium length measurements can be compared only in the experiments in which cells were grown for the same times. All cell lines were grown at 37 °C in a 95% air/5% CO_2_ atmosphere. For three-dimensional (3D) cultures, 1 × 10^4^ MDCK cells were grown as described elsewhere^33^. RPE-1 (CRL4000) and IMCD3 (CRL2123) cells were grown in DMEM/F12 with 10% FBS and primary cilium formation was induced by starving the cells in DMEM/F12 with 0.25% FBS for 24 h.

### DNA constructs and transfection conditions

For CRISPR/Cas9 Cav1 gene editing, the cDNA sequence was analyzed using the Breaking-Cas tool (http://bioinfogp.cnb.csic.es/tools/breakingcas), and the selected target sequences (5′-GGGGCAAATACGTAGACTCCG-3′) for Cav1α KO; (5′-GGTGTACGACGCGCACACCA-3′) for Cav1αβ KO; (5′-CCCGCCGCAGGCAGCTCGTCCGG-3′) for DIA1 KO; (5′- CGCCGTCATGAACTCGCAGCAGG-3′) for INF2 KO; were inserted in the pSpCas9(BB)-2A-GFP plasmid (plasmid 48138; Addgene^75^), which was a gift from F. Zhang (Massachusetts Institute of Technology, Cambridge, MA). GFP-positive cells were sorted after 24 h of transfection and plated. Individual clones were tested by immunofluorescence and immunoblot analysis. The DNA construct expressing Cav1 fused to RFP (plasmid 14434; Addgene^76^ was a gift from A. Helenius (Yale University, CT, USA). To express only the Cav1α-RFP isoform, Met32 and Met36 in the dog Cav1 sequence were mutated to Leu using the QuikChange mutagenesis procedure (Agilent Technologies). The DNA construct expressing Raichu-RhoA^43^, was a gift from E. Kiyokawa (Kyoto University, Kyoto, Japan). Mutagenesis of this plasmid was performed to generate the constitutively active FRET-RhoAV14, changing a G to a V, and the constitutively inactive FRET-RhoAN19, changing a T to a N, as described for Cav1α-RFP. Mutagenesis was performed using the primers (5′-ATGTCTTTCCACAGGCTACATCACCAACAATCACC-3′) for the FRET-RhoAV14 construct and (5′-GGTGATGGAGCCTGTGGAAAGAACTGCTTGCTCATAGTC-3′) for the FRET-RhoAN19 construct. The DNA construct expressing mDia1 fused to GFP^77^ was kindly donated by S. Narumiya (Kyoto University, Kyoto, Japan); the mDia DAD-GFP^49^ was a kind gift from F. Bartolini (Columbia University, New York, NY); the dominant-negative p190RhoGAP mutant was generously donated by K. Burridge (University of North Carolina, Chapel Hill, NC)^44^. All the constructs were verified by DNA sequencing (Macrogen). MDCK cells stably expressing exogenous proteins were generated by transfection and selection with 1 mg/ml G-418 (Santa Cruz Biotechnology, Inc.). Individual clones were screened by fluorescence microscopy. For all constructs, cells were transfected by electroporation in an Amaxa apparatus with the L-005 program. MDCK cells were transfected by electroporation with Amaxa apparatus with 20 nM of control siRNA siCt) HiGC (product 12935–500) or siRNA targeted to RhoA (siRhoA 5′-AACCACUGGUGGGUACAUACACCUCUG-3′). To ensure a high degree of Cav1 depletion, cells were again transfected after overnight incubation, with the same amounts of siRNA siCav1-1 (5′-UGACCAGGUCGAUUUCCUUGGUGUG-3′); and siCav1-2 (5′-CCUUCUGGUUCUGCAAUCACAUCUU-3′) using Lipofectamine 2000. 72 h later, Cav1 knockdown was verified by immunoblotting. RPE-1 and IMCD3 cells were transfected with 20 nM of siRNA negative controls or siRNA targeting to human Cav1 (5′-CUAAACACCUCAACGAUGA-3′) or mouse Cav1 (5′-CACACCAAGGAGAUUGACCUGGUCA-3′), respectively, using Lipofectamine 2000. All siRNAs were from Invitrogen.

### Immunofluorescence analysis

Cells were fixed with 10% formalin (37% formaldehyde solution; Sigma) and permeabilized with 0.2% Triton X-100 and 0.2% SDS for 10 min on ice. They were then blocked, stained with the indicated antibodies, followed by the appropriate secondary antibodies conjugated with Alexa Fluor 488 (excitation at 488 nm and emission collected at 505–530 nm), Alexa Fluor 555 (excitation at 553 nm and emission at 568 nm), Alexa Fluor 594 (excitation at 543 nm and emission at 585–615 nm) or Alexa Fluor 647 (excitation at 633 nm and emission from 650 nm) or with TRITC-phalloidin, and processed as described^78^. For γ-tubulin and acetylated tubulin staining experiments, the γ-tubulin antibody and the corresponding secondary antibody were incubated first and, the same procedure was then repeated for the acetylated tubulin antibody and the secondary IgG2b-specific Alexa-conjugated antibody. Coverslips or Transwell membranes were mounted on glass slides with Fluoromount (Sigma Aldrich). Controls to assess labeling specificity included incubations with control primary antibodies or omission of the primary antibodies. Fluorescence was examined using a confocal laser-scanning microscope LSM800 (ZEISS) with 63x or 100x oil objectives. Brightness and contrast were optimized with Fiji software (National Institutes of Health) or Photoshop (Adobe Systems). LSM images were converted to TIFF format. Quantifications were carried out using Fiji.

### FRET imaging of RhoA activity

MDCK cells stably expressing Raichu-RhoA were grown on chambered coverslips (Ibidi). Five days later, images were captured of living cells maintained at 37 °C in MEM without phenol red, supplemented with 0.25% FBS. All images were captured by a multiphoton LSM710 coupled to an AxioObserver microscope (ZEISS) with a 63x/1.2 Water Plan-Apochromat objective lens. Emission ratio imaging was performed with a 458-nm excitation laser/530-nm emission YFP. FRET-RhoAV14 and FRET-RhoAN19 were transfected for 24 h and analyzed to define the range between the active and inactive states of the biosensor. FRET efficiency was analyzed as previously described^79^ using Fiji software.

### TEM

For TEM analysis, cells were grown on Transwell filters and fixed with 4% paraformaldehyde and 2% glutaraldehyde for 90 min at room temperature. Cell samples were then processed for embedding in Epoxy, TAAB 812 Resin (TAAB Laboratories, Berkshire, UK) following standard procedures. Orthogonal and parallel (from the bottom to the top of the cell) 80-nm-thick ultrathin sections were stained with saturated uranyl acetate and lead citrate by standard procedures. Samples were examined at 80 kV in a Jeol JEM-1010 (Tokyo, Japan) electron microscope. Pictures were taken with a TemCam-F416 (4 K × 4 K) digital camera (TVIPS, Gauting, Germany).

### SEM

Cells were grown on Transwell filters. After 5 days, 4% PFA and 4% glutaraldehyde were added in 1/1 (vol/vol) to MEM containing 5% FBS. After 10 min at room temperature, it was replaced by 2% PFA and 2% glutaraldehyde and incubated for 2 h. Cells were then dehydrated through increasing ethanol concentrations followed by critical-point drying. Dried samples were examined under an FEI VERIOS 460 G4XHR scanning electron microscope at 2.00 kV.

### Pull-down assays for RhoA activity detection and immunoblot analysis

MDCK cells were grown for 5 days and then lysed and subjected to pull-down assays to measure the active GTP-loaded RhoA GTPase, as previously described^80,81^. Briefly, cell lysates were incubated at 4 °C in assay buffer with 10 µg of GST-rhotekin-RBD immobilized on glutathione-sepharose beads. Active RhoA was pulled-down and total RhoA and GST pull-down samples were subjected to SDS-PAGE and transferred onto immobilon membranes (BioRad). After blocking with 5% BSA (wt/vol) and 0.05% (vol/vol) Tween-20 in Tris-buffered saline, membranes were incubated overnight with the indicated antibodies, washed with Tris-buffered saline containing 0.05% Tween 20, and incubated for 30 min with the corresponding secondary antibodies coupled to HRP. The signal was visualized with Clarity Western ECL substrate (BioRad). Band intensities were quantified using Fiji and the results were represented relative to controls.

### Detergent extraction procedure

Cells were lysed for 15 min in 25 mM Tris-HCl (pH 7.2), 150 mM NaCl, and 1% Triton X-100 at 4 °C in the presence of phosphatase and protease inhibitors. The extract was brought to 40% sucrose (w/w) and placed at the bottom of two sequential layers of 30% and 5% sucrose. Gradients were centrifuged to equilibrium, and the soluble fraction and the low-density insoluble membrane fraction were harvested^82^. Equivalent aliquots from the soluble and insoluble fractions were subjected to immunoblot analysis.

### Statistical analysis

Data are expressed as means ± standard deviation (SD). An unpaired Student’s t-test was used to establish the statistical significance of differences between the means using Prism 7.0 software (GraphPad); (*P < 0.05; **P < 0.01; ***P < 0.001). Data were taken from at least three independent experiments.

## Supplementary information

Supplementary information
