# Supplementary material for: Caveolin-1α regulates primary cilium length by controlling RhoA GTPase activity
Source: Sci Rep. 2019 Feb 4;9:1116. doi: 10.1038/s41598-018-38020-5 (PMC6362014; doi:10.1038/s41598-018-38020-5)
Supplement: Supplementary file 1 — Supplementary information [file 41598_2018_38020_MOESM1_ESM.pdf]

Supplementary information

Caveolin-1α regulates primary cilium length by controlling RhoA GTPase activity

Authors: Laura Rangel[1,2], Miguel Bernabé-Rubio[1], Jaime Fernández-Barrera[1], Javier Casares-Arias[1], Jaime Millán[1], Miguel A. Alonso[1]* and Isabel Correas[1,2] *

***Corresponding authors**

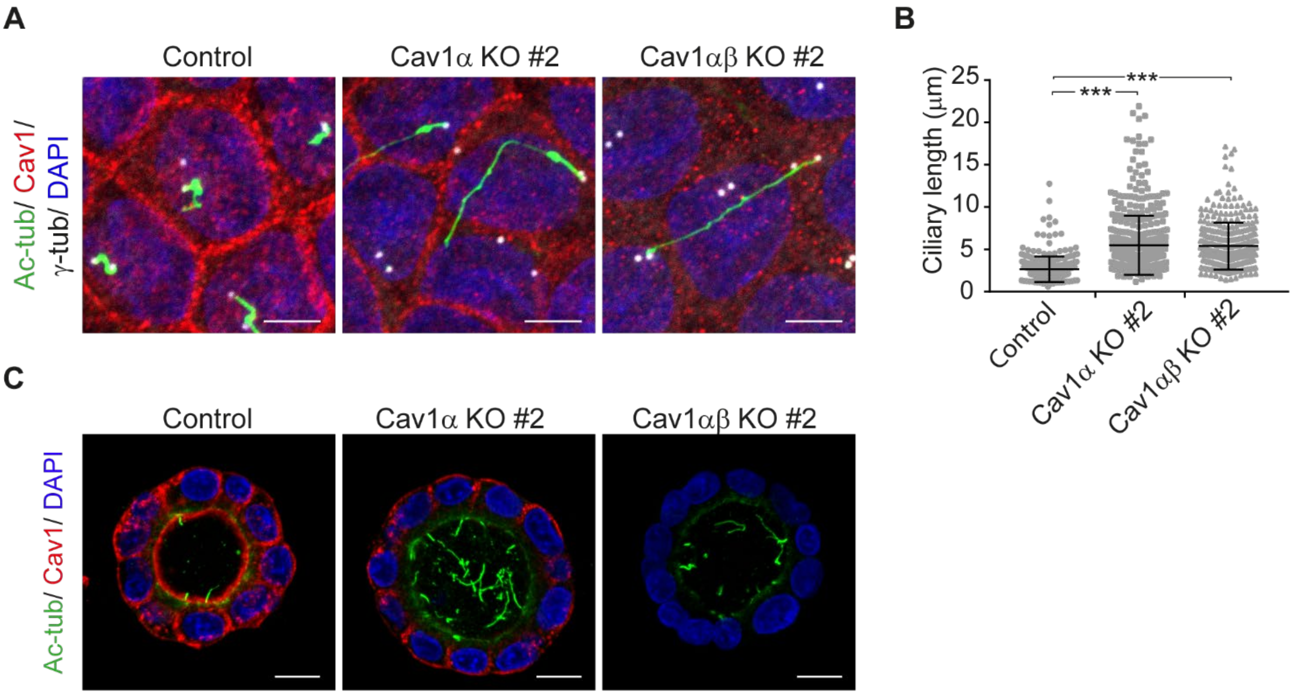

Figure S1. Characterization of other Cav1α and Cav1αβ KO clones. (A, B) Control, and clones #2 of Cav1α KO and Cav1αβ KO shown in Fig.2 were cultured in Transwell inserts for 5 days and stained for acetylated tubulin, Cav1, γ-tubulin and nuclei (A). The scatter-plot represents cilium lengths measured in μm; at least 650 cells were analyzed for each condition (B). **(C)** Cells were cultured in 3D for 5 days and stained for acetylated tubulin, Cav1 and nuclei. Data were pooled from three independent experiment and are presented as means ± SD. ****P*< 0.001; ns, non-significant. Scale bars, 5 μm (A); 10 μm (C).

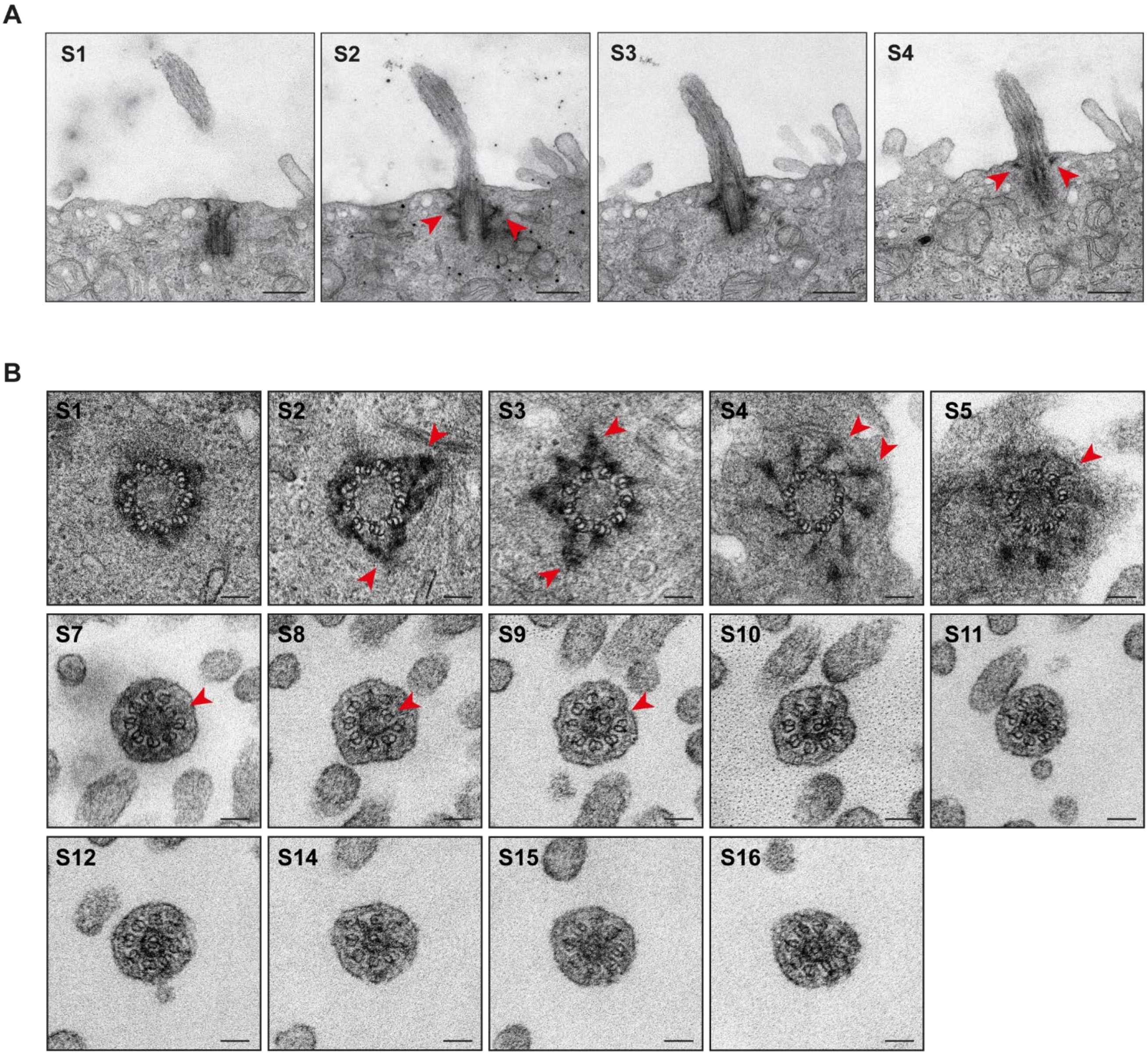

Figure S2. Cav1α KO cilium ultrastructure. (A) Serial sections of a Cav1α KO primary cilium. Serial longitudinal sections (S1 to S4) are numbered from front to back. Arrowheads point to the basal feet in S2 and to the transition fibers in S4. **(B)** Representative cross-sections of a primary cilium of Cav1α KO cells. Serial sections are numbered from the basal body (S1 to S4) to the most distal observed section of the axoneme (S5 to S16). Arrowheads indicate the basal feet in S2 and S3, the transition filaments in S4, Y-links in S5, and the microtubule doublet that moves to the center of the axoneme (S7 to S9). Scale bars, 0.4 μm (A); 0.09 μm (B).

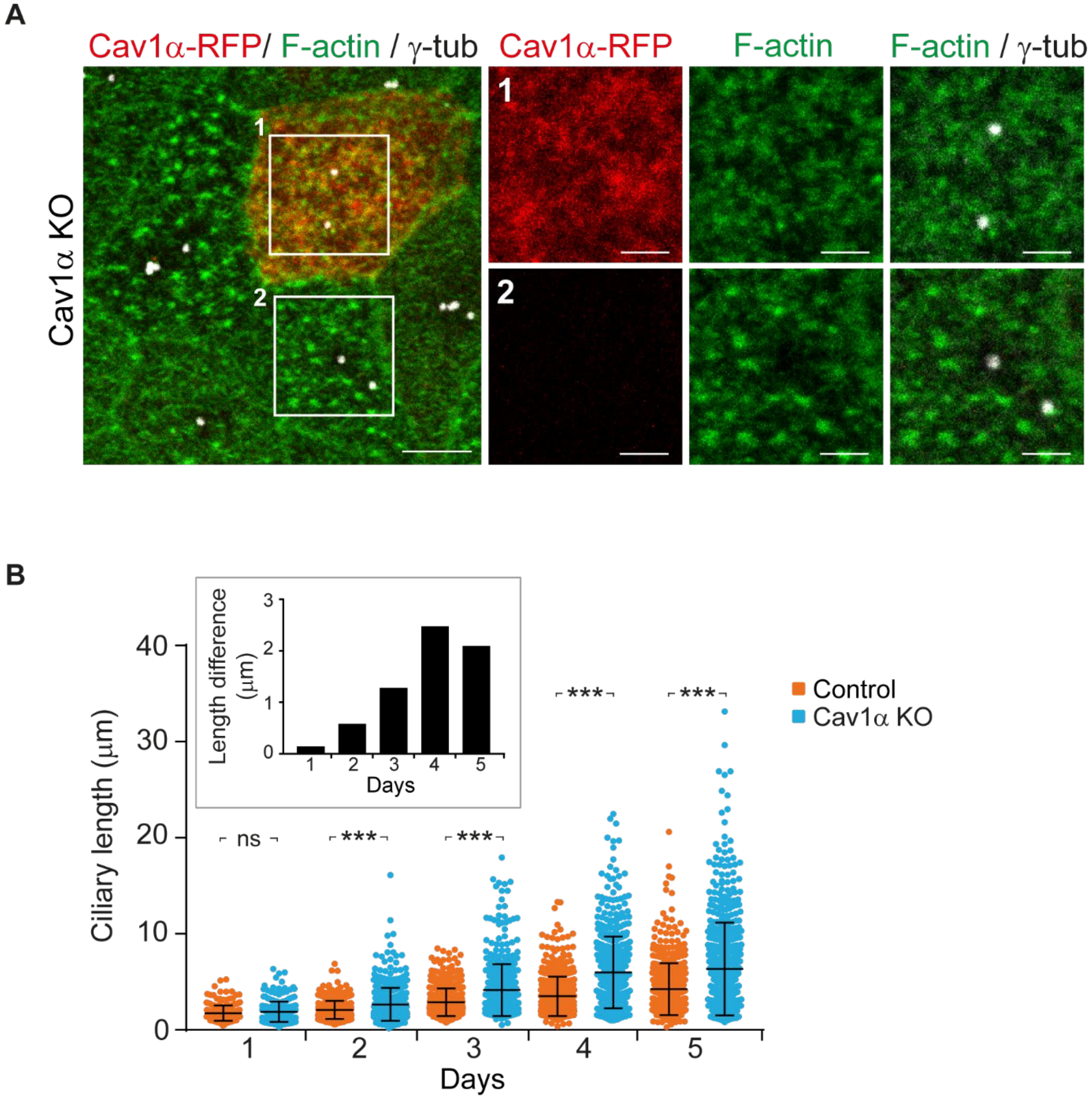

Figure S3. Exogenous Cav1α rescues apical actin meshwork distribution. (A) Cav1α KO cells were transiently transfected with Cav1α-RFP. After 4 days, cells were fixed and stained for F-actin and γ-tubulin. The boxed regions of a transfected (1) and an untransfected (2) cell are shown enlarged in the right panels as separate channels. Scale bar, 5 µm (left panel); 2 µm (right panels). **(B)** Cells were cultured in Transwell inserts and fixed every day during 5 days for immunofluorescence analysis. The scatter-plot represents ciliary length of control and Cav1α KO cells at the indicated times. The inset shows the mean difference between control and Cav1α KO ciliary length at the indicated times. Data were pooled from three independent experiments and are presented as means ± SD. ***$P < 0.001$; ns, non-significant.

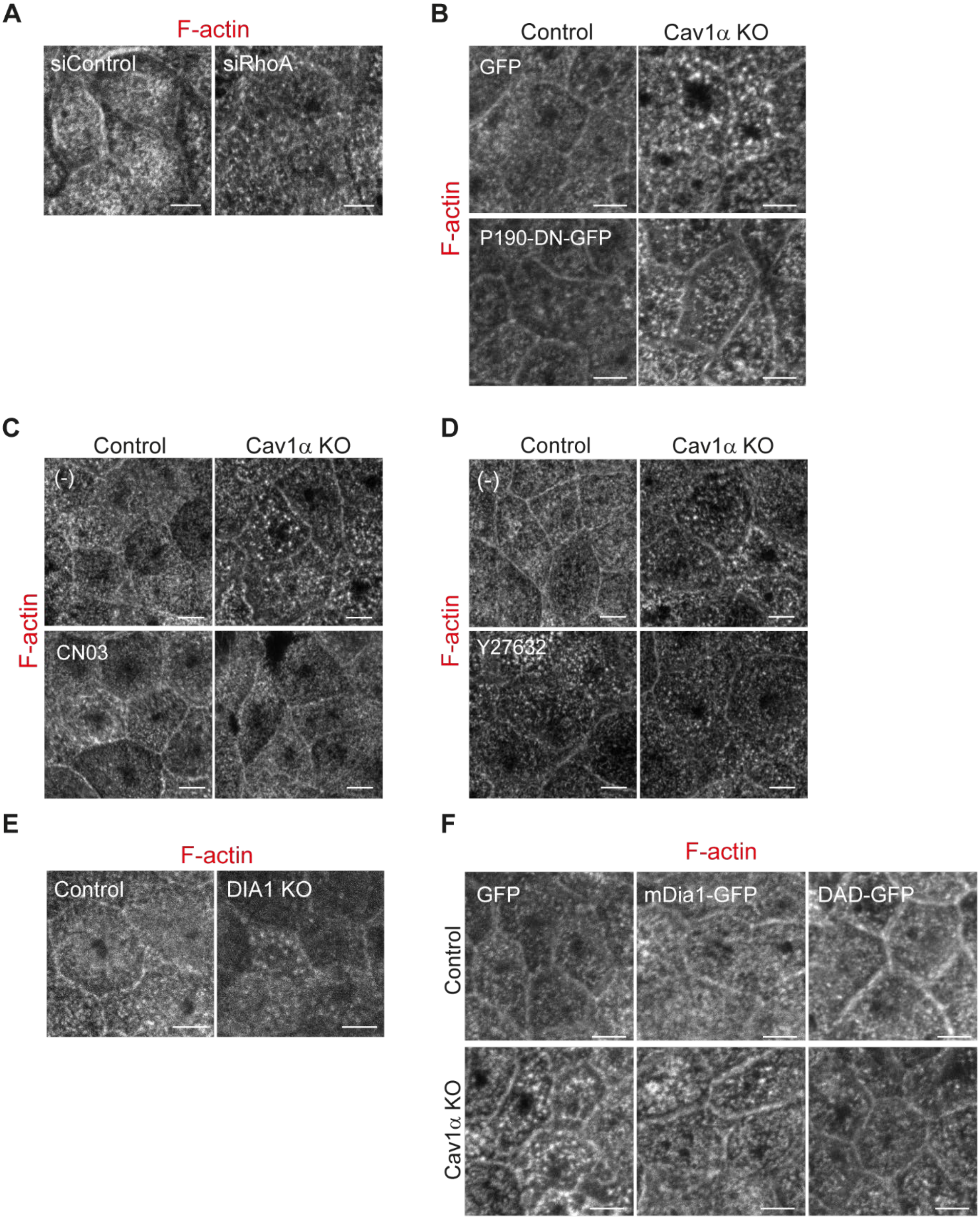

Figure S4. F-actin staining corresponding to the images shown in Figures 6 and 7. F-actin staining of Figures 6E **(A)**, 6G **(B)**, 6I **(C)**, 7A **(D)**, 7D **(E)** and 7F **(F)**. Scale bars: 5 μm.

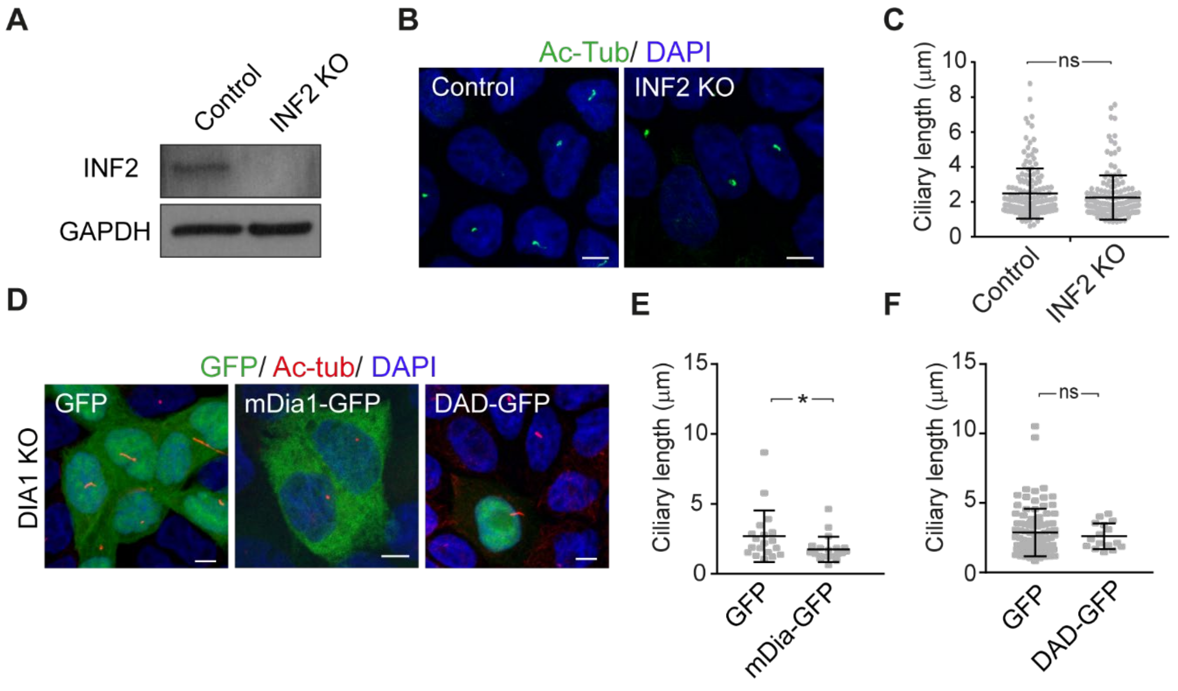

Figure S5. Primary cilium length is not altered in INF2 KO cells. (A) Control and INF2 KO MDCK cells were analyzed by immunoblotting for INF2. GAPDH was used as a protein-loading control. **(B, C)** Control and INF2 KO cells were grown for 5 days and stained for acetylated tubulin and nuclei (B). The scatter-plot represents ciliary length measured in µm; more than 500 cells were analyzed for each condition (C). **(D-F)** DIA1 KO cells were transiently transfected with the indicated constructs. After 72 h, cells were fixed and stained for acetylated tubulin and nuclei (D). Scatter-plots represent cilium lengths measured in µm; at least 75 cells were analyzed for each condition (E, F). Data in C, E and F were pooled from at least three independent experiments and are represented as the mean ± SD. *P<0.05; ns, non-significant. Scale bars: 5 µm.

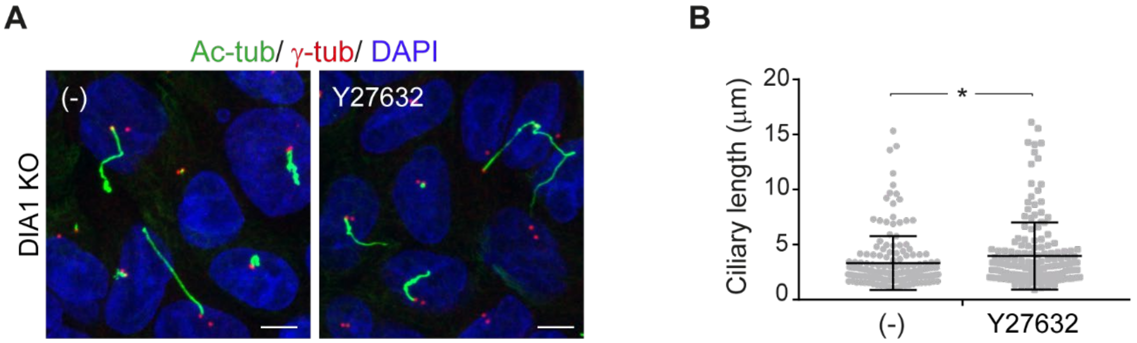

Figure S6. ROCK and DIA1 act in coordination to regulate cilium lengthening. (A, B) DIA1 KO cells were untreated (-) or treated with 10 µM Y27632 for 24 h and stained for acetylated tubulin, γ-tubulin and nuclei (A). The scatter-plot represents ciliary length measured in µm for each condition. More than 600 cells were analyzed for each experimental condition (B). Data were pooled from three independent experiments and are represented as the mean ± SD. $^*P<0.05$. Scale bars: 5 µm.

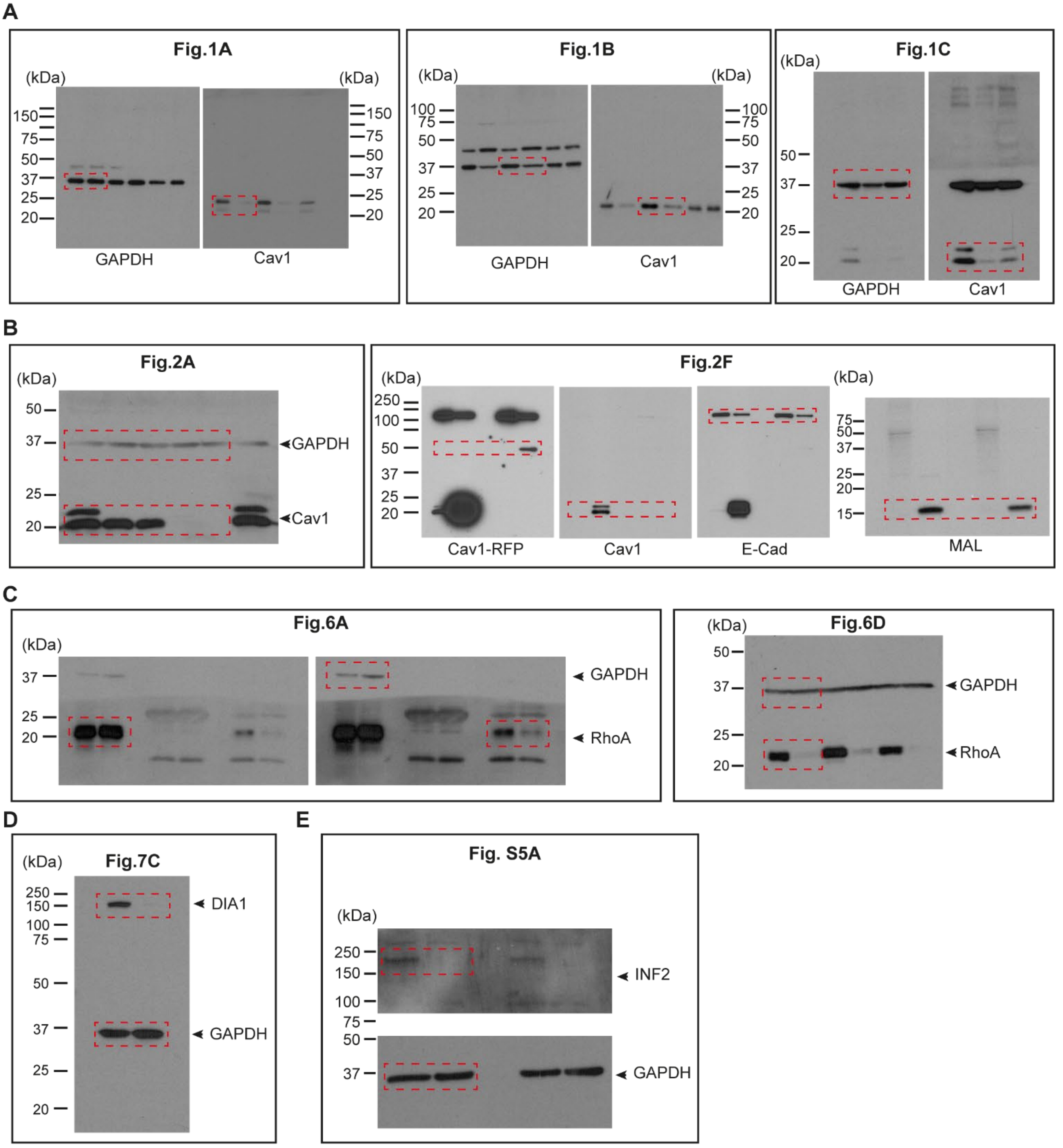

Figure S7. Full-length blots corresponding to Figures 1, 2, 6, 7 and S5. (A) Full-length blots corresponding to figures 1A and 1B. Two exposures are shown in the blot of figure 1C which was cut between 25 and 37 kDa bands and aligned before exposure. (B) Full length blots are shown corresponding to figure 2. The membrane of figure 2A was cut between 25 and 37 kDa bands and aligned before exposure. Different exposures are shown of the blot corresponding to figure 2F, the membrane was cut between 25-37 kDa and at the 75 kDa bands and aligned before exposure. The blot of MAL becomes from a more concentrated gel with the same cell extracts. (C) Blots corresponding to figure 6. In the blot of figure 6A, two exposures are shown. The membranes corresponding to figure 6A and 6D were cut between 25 and 37 kDa bands and aligned before exposure. (D) Blot corresponding to figure 7C, the membrane was cut between 25 and 37 kDa bands and aligned before exposure. (E) Blot corresponding to supplementary figure 5A, the membrane was cut at the 75 kDa band.
